# Integrated urban water management by coupling iron salt production and application with biogas upgrading

**DOI:** 10.1038/s41467-023-42158-w

**Published:** 2023-10-12

**Authors:** Zhetai Hu, Lanqing Li, Xiaotong Cen, Min Zheng, Shihu Hu, Xiuheng Wang, Yarong Song, Kangning Xu, Zhiguo Yuan

**Affiliations:** 1 Australian Centre for Water and Environmental Biotechnology, The University of Queensland, St Lucia, QLD 4072 Australia; 2 State Key Laboratory of Urban Water Resource and Environment, Harbin Institute of Technology, Harbin, 150090 PR China; 3 School of Environment, Harbin Institute of Technology, Harbin, 150090 PR China; 4 Beijing Key Laboratory for Source Control Technology of Water Pollution, Collage of Environmental Science and Engineering, Beijing Forestry University, Beijing, 100083 China; 5 School of Energy and Environment, City University of Hong Kong, Hong Kong SAR, China

**Keywords:** Civil engineering, Pollution remediation, Chemical engineering, Carbon capture and storage

## Abstract

Integrated urban water management is a well-accepted concept for managing urban water. It requires efficient and integrated technological solutions that enable system-wide gains via a whole-of-system approach. Here, we create a solid link between the manufacturing of an iron salt, its application in an urban water system, and high-quality bioenergy recovery from wastewater. An iron-oxidising electrochemical cell is used to remove CO_2_ (also H_2_S and NH_3_) from biogas, thus achieving biogas upgrading, and simultaneously producing FeCO_3_. The subsequent dose of the electrochemically produced FeCO_3_ to wastewater and sludge removes sulfide and phosphate, and enhances sludge settleability and dewaterability, with comparable or superior performance compared to the imported and hazardous iron salts it substitutes (FeCl_2_, and FeCl_3_). The process enables water utilities to establish a self-reliant and more secure supply chain to meet its demand for iron salts, at lower economic and environmental costs, and simultaneously achieve recovery of high-quality bioenergy.

## Abstract (web summary)

Effective urban water management requires technological solutions that enable system-wide gains via a holistic approach. Here, authors propose an integrated system where an iron-oxidising electrochemical cell upgrades biogas while producing FeCO_3_ and subsequently uses the salt in wastewater treatment.

## Introduction

Iron salts in various forms (FeCl_2_, FeCl_3_, FeSO_4_ and Fe_2_(SO_4_)_3_) are widely used in urban water management for a variety of purposes^1–3^. Most drinking water treatment plants rely on the use of iron- (or aluminum-) based coagulants for the removal of turbidity and natural organic matter^4, 5^. Similarly, the addition of iron salts to sewer networks is widely applied to combat hydrogen sulfide (H_2_S) induced sewer corrosion and odor^6–8^, a notorious and multi-billion-dollar problem in sewer management^9,10^. Further, many wastewater treatment plants (WWTPs) rely on the dosing of iron- (or aluminum-) based salts for the removal of phosphate^11,12^, and for improving sludge settleability and dewaterability^13,14^. Lastly, iron salts are also dosed to anaerobic digesters for reducing H_2_S in biogas^15,16^. These broad applications of iron salts lead to their consumption in large quantities. Indeed, iron salts represent a significant fraction of coagulants and flocculants consumed by the water industry, which had a global market of USD 6.4B in 2018 and is expected to reach USD 8.5B in 2023^17^.

Iron salts currently used by the water industry are manufactured as a by-product of metallurgical processes. For example, the iron salts supplied in Australia are produced in the steel pickling process. Hydrochloric acid (HCl) or sulfuric acid (H_2_SO_4_) is used to remove iron oxides at the surface of steel, resulting in an acidic spent pickling liquor containing FeCl_2_ or FeSO_4_. These ferrous salts can be further converted to ferric salts, if needed, via the addition of a strong oxidant such as chlorine (Cl_2_) or peroxide (H_2_O_2_)^18^. In some other parts of the world, FeCl_2_ or FeSO_4_ are produced from titanium ores containing iron, as a by-product in titanium dioxide (TiO_2_) production, again involving the use of Cl_2_/HCl or H_2_SO_4_^19^. Iron salts are also produced from iron ore, or by dissolving iron using HCl or H_2_SO_4_^20^.

With the current supply chain, the sources of iron salts are in most cases a long way away from where they are required, resulting in the need for long-distance transport. This increases the costs and environmental footprint of the chemical supply and poses significant occupational health and safety (OH&S) challenges due to the hazardous and corrosive nature of these chemicals. The current supply chain is susceptible to many factors, e.g., both UK and Germany water utilities are currently in shortage of iron salts due to supply chain interruptions, which had forced the local authorities to allow the discharge of partially treated sewage to the environments. It is of strategic importance for the water industry to establish local and environmentally friendly iron salt supplies that have higher supply chain security.

There is currently an on-going paradigm shift in wastewater management from pollutant removal to resource recovery. The recovery of bioenergy, in the form of biogas, is now widely implemented. Biogas produced at a WWTP is currently almost exclusively used locally for thermal and electrical energy generation^21–24^. However, limited by the relatively low electricity price and low value of thermal energy at most places, the value of biogas thus derived is generally low, especially considering the significant capital and maintenance costs associated with the gas engines^25^. The high-value uses of biogas, for example as a transport fuel or for injection into the natural gas grids^26,27^, require the removal of CO_2_, which typically constitutes 30 − 50% of biogas^28^. Various physical and chemical processes have been developed and applied to efficiently remove CO_2_ from biogas thus achieving biogas upgrading^28–31^. However, they are often energy-inefficient and most leave behind materials requiring disposal or regeneration, potentially causing secondary pollution^29^. For example, CO_2_ absorption using amine solutions results in degraded solvent that are toxic to both humans and the environment^32^.

In this work, we propose and demonstrate an electrochemical method for manufacturing iron salts, a solution that effectively addresses two challenges simultaneously. The proposed method is fundamentally different from the existing method of chemical iron salts production^13,33^. The proposed method facilitates the establishment of a local iron salts supply chain and simultaneously broadens the range of biogas applications. Specifically, an iron-oxidizing electrochemical process is introduced to remove CO_2_ from biogas, thus upgrading biogas. Concomitantly, as a CO_2_ sink, FeCO_3_ is produced, which can be introduced to an urban water system as a substitute of the currently used iron salts, with comparable or superior performance. Mass balance assessment shows that the amount of FeCO_3_ produced at a WWTP via this pathway meets the demand for iron salts by the catchment collecting and transporting wastewater to the plant. The economic and life-cycle assessments show that the supply pathway proposed in this study is more cost effective and more environmentally friendly than the current supplies.

## Results

### Electrochemical CO_2_ removal from biogas and FeCO_3_ production

The CO_2_ removal tests were conducted in an electrochemical cell modified from a glass bottle, with two iron plates as the electrodes (Supplementary Fig. 1). NaCl at 2 g/L, sparged with the feed gas for about 30 min to strip dissolved oxygen, was used as the electrolyte. The feed gas contained CO_2_ at ~40%, CH_4_ (or N_2_ as a non-explosive surrogate of CH_4_) at ~60%, and trace levels of H_2_S at ~900 ppmv and NH_3_ at ~270 ppmv in some tests.

**Fig. 1:**
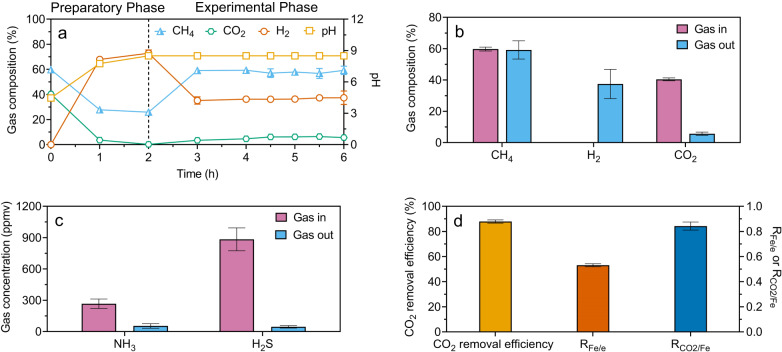
Reactor performance in the tests at a pH set-point of 8.5. **a** Contents of CH_4_, H_2_, and CO_2_ in the headspace along with the electrolyte pH. **b** Average CH_4_, H_2_, and CO_2_ concentrations in the feed and upgraded gas. **c** Average NH_3_ and H_2_S concentrations in the feed and upgraded gas. **d** The Fe-to-electron ratio (R_Fe/e_) and the CO_2_-to-Fe ratio (${{{\mbox{R}}}}_{{{{\mbox{CO}}}}_{2}/{{\mbox{Fe}}}}$). The vertical dotted line in (a) represents the start of continuous gas feeding (i.e. the commencement of experimental phase). All values are means ± standard in deviations of triplicate tests.

Each 6 h test comprised a 2 h preparatory phase followed by a 4 h experimental phase (Fig. 1a, Supplementary Figs. 3−6). A current was supplied in the preparatory phase to produce Fe^2+^ at the anode $({{{{{\rm{Fe}}}}}}\to {{{{{{\rm{Fe}}}}}}}^{2+}+2{{{{{{\rm{e}}}}}}}^{-})$ and OH^−^ at the cathode $(2{{{\mbox{H}}}}_{2}{{\mbox{O}}}+2{{{{{{\rm{e}}}}}}}^{-}\to {{2{{\mbox{OH}}}}^{-}+{{{{{\rm{H}}}}}}}_{2})$. In the absence of gas feeding, dissolved inorganic carbon (CO_2_, ${{{\mbox{HCO}}}}_{3}^{-}$, and ${{{\mbox{CO}}}}_{3}^{2-}$) as well as CO_2_ in the reactor headspace, resulting from the initial gas sparging, were removed (Fig. 1a, Supplementary Figs. 3−6) via reactions ${{{\mbox{CO}}}}_{2}+{{{\mbox{H}}}}_{2}{{\mbox{O}}}\to {{{\mbox{HCO}}}}_{3}^{-}+{{{\mbox{H}}}}^{+}\to \,{{{\mbox{CO}}}}_{3}^{2-}+2{{{\mbox{H}}}}^{+}$; ${{{\mbox{Fe}}}}^{2+}+{{{\mbox{CO}}}}_{3}^{2-}\to {{{\mbox{FeCO}}}}_{3}$; $2{{{\mbox{H}}}}^{+}+2{{{\mbox{OH}}}}^{-}\to {2{{\mbox{H}}}}_{2}{{\mbox{O}}}$. The continued current supply following CO_2_ depletion led to pH elevation to the pre-selected set-point (i.e. 7.5, 8.0, 8.5, or 9.0) due to the on-going production of hydroxide (Fig. 1a, Supplementary Figs. 3−6).

In the subsequent experimental phase, the feed gas was continuously fed to the cell. The continuous CO_2_ removal resulted in an upgraded gas containing substantially lower level of CO_2_ (e.g. 6.1 ± 0.1% in Fig. 1a). Concomitantly, H_2_ produced in the cathodic reaction evolved into the headspace replacing CO_2_ removed (Fig. 1a, b, Supplementary Figs. 3−6). The current was manually adjusted to keep the electrolyte pH at the set-point (8.5 in Fig. 1, Supplementary Figs. 5, 7, and at other pH levels in Supplementary Figs. 3, 4, and 6). The concentrations of NH_3_ and H_2_S in the gas were reduced from 267.5 ± 26.1 ppmv and 884.1 ± 63.5 ppmv to 54.3 ± 12.3 ppmv and 46.2 ± 6.8 ppmv, respectively (Fig. 1c), along with CO_2_ removal. In contrast, CH_4_ (or N_2_) in the feed gas directly evolved into the headspace due to their low solubilities (Fig. 1a, b, Supplementary Figs. 2−7).

At pH 8.5, the ratio between the Fe oxidized and electrons transferred (R_Fe/e_) was 0.52 ± 0.01 (mole/mole), suggesting the majority of electrons transferred were produced from Fe oxidation to Fe^2+^. The ratio between CO_2_ removed and Fe oxidized $({{{\mbox{R}}}}_{{{{{{\rm{CO}}}}}}_{2}/{{{{\rm{Fe}}}}}})$ was 0.84 ± 0.03 (mole/mole), suggesting the majority of Fe^2+^ produced was used for CO_2_ removal (Fig. 1d). Overall, the results demonstrate the feasibility of CO_2_, H_2_S, and NH_3_ removal from biogas using an iron-oxidizing electrochemical cell.

**Fig. 2:**
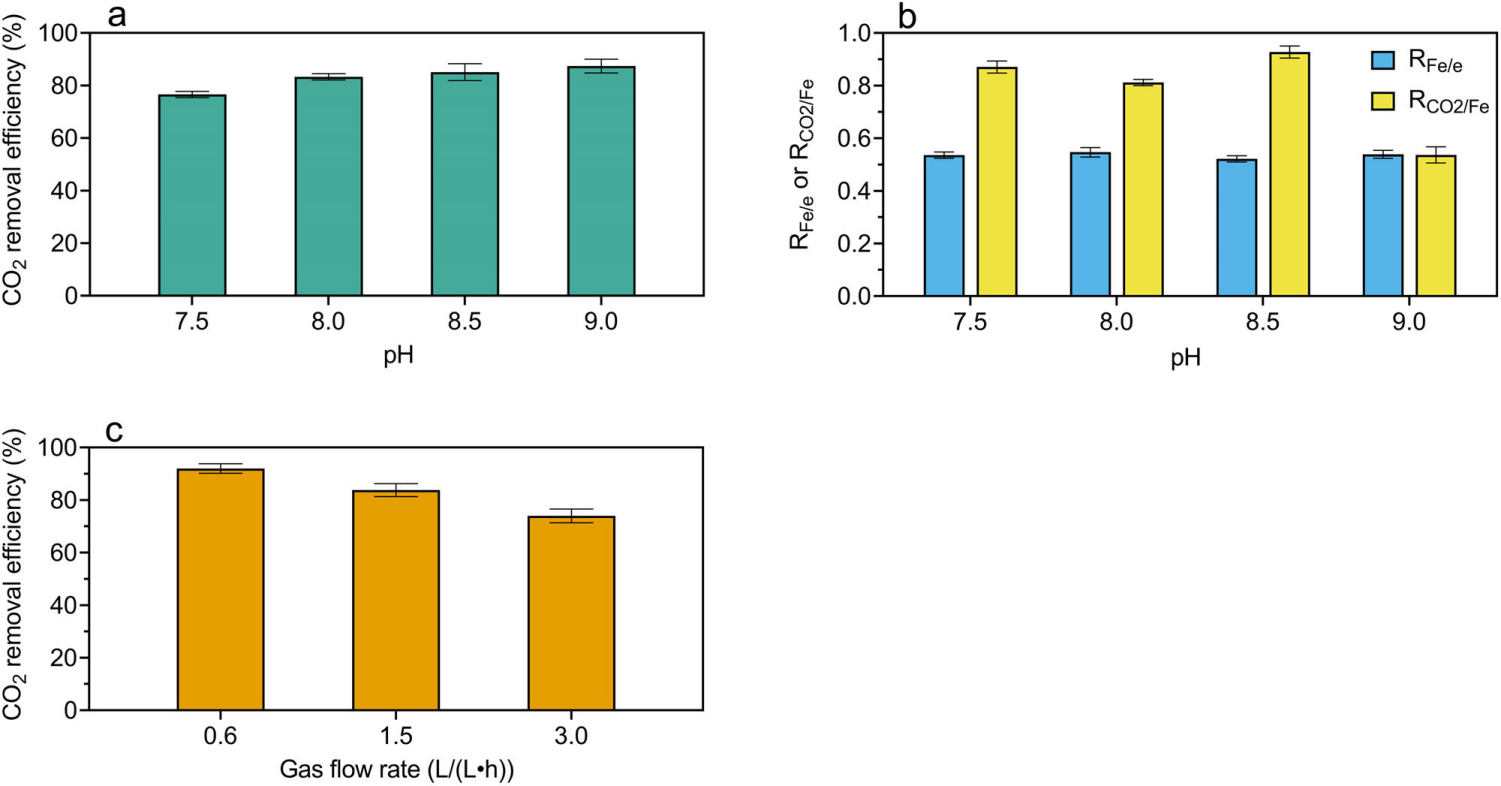
Effects of pH and gas flow rate on cell performance. **a** CO_2_ removal efficiency in the tests at pH 7.5, 8.0, 8.5, and 9.0. **b** R_Fe/e_ and ${{{\mbox{R}}}}_{{{{{{\rm{CO}}}}}}_{2}/{{{{\rm{Fe}}}}}}$ in the tests at pH 7.5, 8.0, 8.5, and 9.0. **c** CO_2_ removal efficiency in the tests with gas flow rates of 0.6, 1.5, and 3.0 L/(L‧h) at a pH set-point of 8.5. All values are means ± standard deviations of triplicate tests.

The cell performance is strongly pH dependent. Lower headspace CO_2_ contents were achieved with the increase of pH (Fig. 2a, Supplementary Figs. 3–6). However, ${{{\mbox{R}}}}_{{{{{{\rm{CO}}}}}}_{2}/{{{{\rm{Fe}}}}}}$ decreased sharply when pH increased from 8.5 to 9.0 (Fig. 2b, Supplementary Table 1), indicating that a substantial fraction of ferrous ions produced was not combined with carbonate at pH 9.0, likely due to the formation of Fe(OH)_2_ as an additional precipitate. Overall, pH 8.5 appears to be a favorable condition with relatively high CO_2_ removal efficiency (i.e. 85.1 ± 0.4%) and ${{{\mbox{R}}}}_{{{{{\rm{Fe}}}}}/{{{{\rm{e}}}}}}$ (i.e. 0.52 ± 0.01), and satisfactory ${{{\mbox{R}}}}_{{{{{{\rm{CO}}}}}}_{2}/{{{{\rm{Fe}}}}}}$ (i.e. 0.84 ± 0.03).

The gas flow rate also impacted the CO_2_ removal efficiency (Fig. 2c). The CO_2_ concentration in the upgraded gas increased with the gas flow rate from 3.2 ± 0.3% at 0.6 L/(L‧h), to 6.2 ± 0.1% at 1.5 L/(L‧h), and then to 9.5 ± 0.5% at 3.0 L/(L‧h). An increase in the gas flow rate reduces the gas residence time^34^, which decreases the CO_2_ reaction time and reduces the CO_2_ removal efficiency. The results suggest that a high level of CO_2_ removal is possible by designing the reactor and the gas supply so that a satisfactory gas retention time and gas transfer rate is achieved.

The FeCO_3_ produced, called E-FeCO_3_ hereafter to be distinguished from the commercially available FeCO_3_ (C-FeCO_3_) that will later also be used in experimental studies, exists as solids in a slurry. The average particle size in the slurry produced in the cell is in the micron range with the D_10_, D_50_, and D_90_ values being 6.9 ± 0.6, 20.1 ± 2.3, and 46.7 ± 3.2 μm, respectively (Supplementary Fig. 8a, b). The particle size was measured as it likely influences the efficacy of E-FeCO_3_ to react with sulfide or phosphate when added to wastewater or sludge, due to e.g. surface limitations or solids settling. Anaerobic storage of the slurry for up to 4 weeks did not significantly (*p* = 0.73) change the particle size distributions (Supplementary Fig. 8a, b). The particles, freshly produced or stored for up to 4 weeks, remained in suspension under turbulent conditions simulating those in sewers (Supplementary Fig. 8c). This means that the particles would remain suspended in sewage after in-sewer dosing, a desirable property for its in-sewer use.

Three crystalline iron species in the E-FeCO_3_ slurry were identified to be siderite (FeCO_3_), goethite (α-FeO(OH)), and hematite (Fe_2_O_3_) (Supplementary Fig. 9). Among these, FeCO_3_ is the only compound containing Fe^2+^, thus the measured fraction of Fe^2+^ in total Fe (86.2 ± 3.9%) represents the fraction of FeCO_3_ in all Fe-containing compounds. This is consistent with the measured ratio between CO_2_ removed and Fe oxidized $({{{\mbox{R}}}}_{{{{{{\rm{CO}}}}}}_{2}/{{{{\rm{Fe}}}}}})$, which is 0.84 ± 0.03.

### Application of E-FeCO_3_ to wastewater and sludge management

**Fig. 3:**
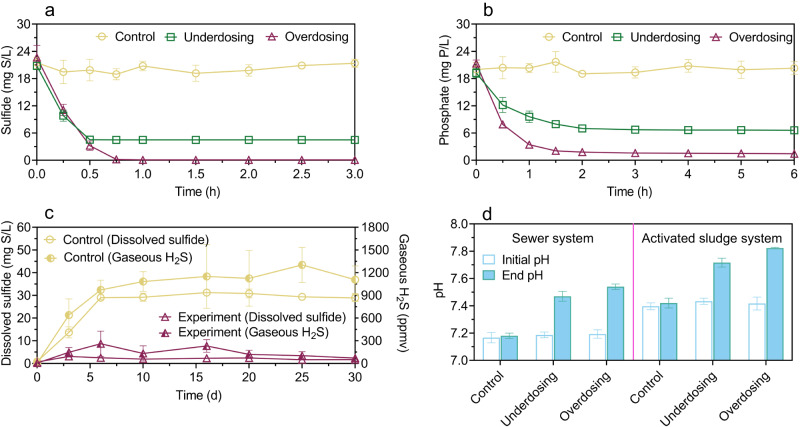
Application of FeCO_3_ slurry to wastewater, activated sludge, and anaerobic digester. **a** Sulfide removal from sewage. **b** Phosphate removal in aerated activated sludge, **c** Sulfide removal in an anaerobic sludge digester, **d** pH at the end of each batch test in (**a**) and (**b**). The FeCO_3_ slurry added was freshly produced at pH 8.5. All values are means ± standard deviations of triplicate tests.

The E-FeCO_3_ slurry was added to anaerobic sewage, aerated activated sludge, and an anaerobic sludge digester to test its potential to remove sulfide and phosphate, despite Fe^2+^ is in precipitates rather than as a dissolved ion. Dosed to anaerobic sewage, the E-FeCO_3_ slurry quickly reduced the dissolved sulfide concentration in 0.5 h (Fig. 3a). The ratio between the sulfide removed and the Fe dosed, determined from the results in the underdosing tests, was 0.51 ± 0.04 g S/g Fe (Fig. 3a). Meanwhile, the wastewater pH was raised by 0.3 unit (Fig. 3d), caused by the release of carbonate from the E-FeCO_3_ slurry. An increase in pH is favorable for sulfide and Fe^2+^ precipitation^3,35^. Indeed, the dissolved sulfide concentration reduced to 0.08 ± 0.02 mg S/L when the E-FeCO_3_ slurry was overdosed (Fig. 3a). The dosing of the E-FeCO_3_ slurry to an anaerobic sludge digester controlled dissolved sulfide at 1.8 ± 0.4 mg S/L, compared to 30.5 ± 1.9 mg S/L in control (Fig. 3c), with H_2_S in biogas reduced from 1171.8 ± 269.2 ppmv (in control) to 85.7 ± 55.1 ppmv (in the experimental digester). Biogas production was not affected by E-FeCO_3_ dosing (Supplementary Fig. 10).

Controlling effluent phosphate concentration at a low level is essential for impeding eutrophication. When added to aerated activated sludge, the E-FeCO_3_ slurry significantly (*p* < 0.01) reduced the phosphate concentration within an hour (Fig. 3b). The ratio between phosphate removed and Fe dosed was 0.56 ± 0.02 g P/g Fe, determined from the results in the underdosing tests (Fig. 3b). A phosphate concentration of 1.62 ± 0.09 mg P/L was achieved in the overdosing tests (Fig. 3b). Meanwhile, the sludge pH in the experimental reactors was significantly (*p* < 0.01) higher than that in control (Fig. 3d).

The E-FeCO_3_ slurry, after storage for 1, 2 and 4 weeks, showed similar sulfide removal performance to that of the fresh slurry, with the majority of sulfide removed within 1 h (to below 0.1 mg S/L). The sulfide to Fe ratios were 0.46 ± 0.02 g S/g Fe, 0.51 ± 0.03 g S/g Fe, and 0.48 ± 0.02 g S/g Fe, respectively (Supplementary Fig. 11). The sewage pH also significantly (*p* < 0.01) increased from ~7.2 to ~7.5.

**Fig. 4:**
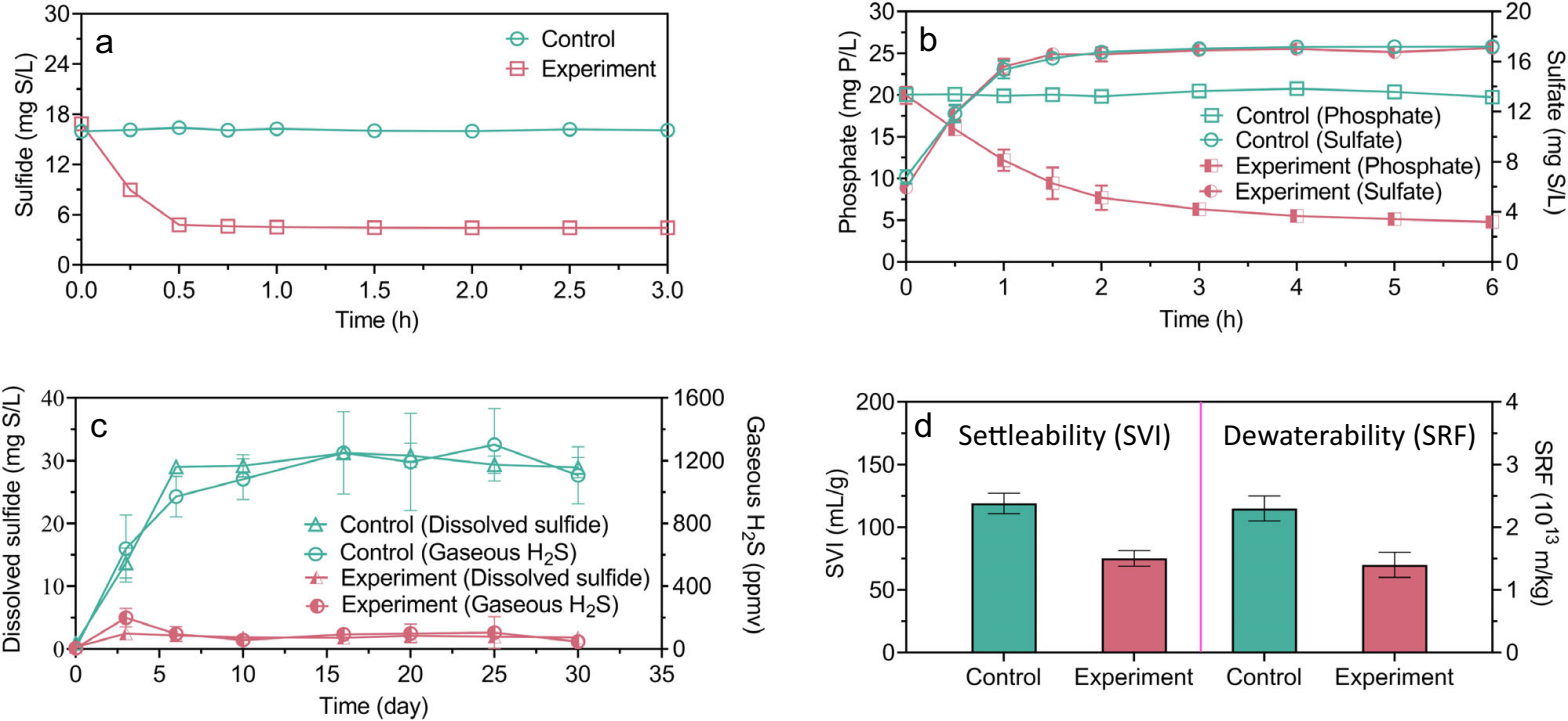
Flowing-on effects of in-sewer dosing of E-FeCO_3_ slurry on downstream wastewater and sludge treatment processes. **a** Sulfide control in sewer. **b** Phosphate removal in aerated activated sludge. **c** Sulfide control in an anaerobic sludge digester. **d** Improvement to sludge settleability (sludge volume index, SVI) and dewaterability (specific resistance to filtration, SRF). All values are means ± standard deviations of triplicate tests.

Sewer networks, wastewater treatment processes, and anaerobic sludge digesters are interconnected in an urban wastewater system. The impacts of E-FeCO_3_ dosing to sewer networks on the performance of downstream biological wastewater treatment and anaerobic sludge digestion processes were investigated via a series of batch experiments by adding E-FeCO_3_-dosed sewage to aerated sludge, which was subsequently added to an anaerobic sludge digester. In-sewer E-FeCO_3_ dosing resulted in phosphate removal in the aerated sludge at a ratio of 0.51 ± 0.09 mg P/mg Fe (Fig. 4b), following sulfide removal in the sewer reactor (Fig. 4a). This was likely due to the oxidation of FeS particles formed in the anaerobic sewer in the aerated activated sludge, as indicated by the increased sulfate concentration, resulting in a flowing-on effects of phosphate precipitation with the regenerated iron (Fig. 4b). Nitrification by the aerated sludge was not impacted by the wastewater amendments with the E-FeCO_3_ slurry (Supplementary Fig. 12). The anaerobic digestion of the activated sludge receiving the E-FeCO_3_-dosed sewage had negligible sulfide accumulation in the digester and biogas, despite complete sulfate reduction, in clear contrast to the control (Fig. 4c). These results suggest that the iron originally dosed to the sewer reactor had a further flowing-on effects of sulfide control in the digester. The methane production performance was not impacted (Supplementary Fig. 13). The settleability and dewaterability of the sludge receiving E-FeCO_3_ amended sewage were also found to be significantly (*p* < 0.01) improved by 36.9 ± 2.7% and 39.1 ± 4.5%, respectively (Fig. 4d).

### Comparison of E-FeCO_3_ with other iron salts in wastewater and sludge management performance

**Fig. 5:**
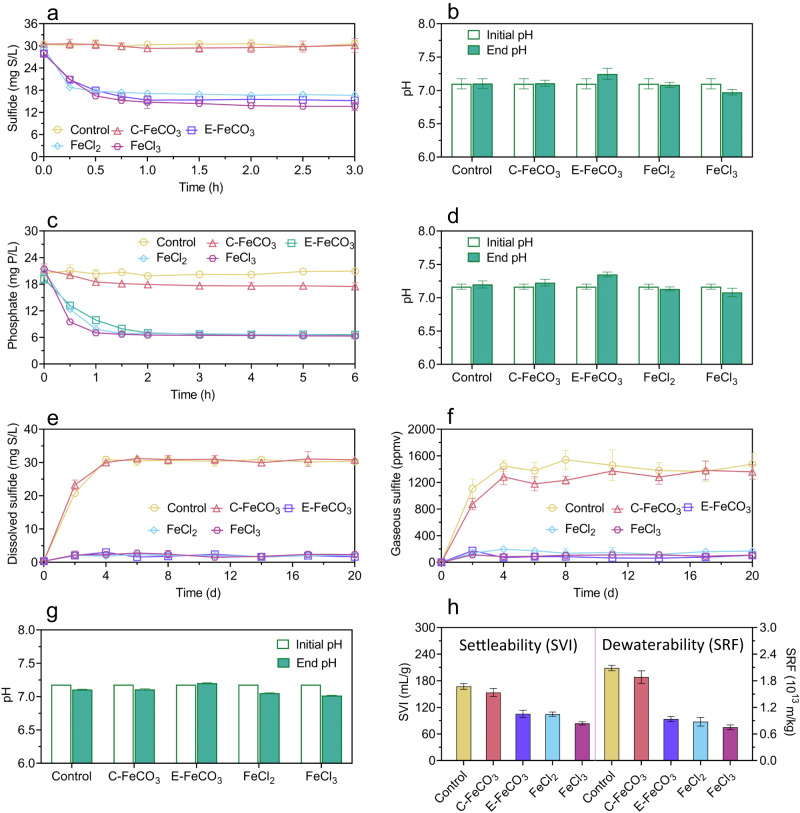
Comparison of E-FeCO_3_ with other iron salts in wastewater and sludge management performance. **a** Sulfide control in sewer (underdosing). **b** pH at the beginning and end of each test in sewer (underdosing). **c** Phosphate removal in aerated activated sludge (underdosing). **d** pH at the beginning and end of each test in aerated activated sludge (underdosing). **e** Dissolved sulfide control in an anaerobic sludge digester. **f** Gaseous hydrogen sulfide (H_2_S) control in an anaerobic sludge digester. **g** pH at the beginning and end of each test in an anaerobic sludge digester. **h** Improvement to sludge settleability (sludge volume index, SVI) and dewaterability (specific resistance to filtration, SRF). All values are means ± standard deviations of triplicate tests.

The experimental results provided compelling evidence for the efficacy of E-FeCO_3_ in removing sulfide and phosphate, as well as its ability to improve sludge settleability and dewaterability. Further, the performance of E-FeCO_3_ was compared with the C-FeCO_3_, FeCl_2_, and FeCl_3_. C-FeCO_3_ displayed negligible ability to remove sulfide or phosphate from wastewater/sludge, and limited ability to improve sludge settleability and dewaterability (Fig. 5). This could be attributed to its more stable crystalized structure in larger particles (Supplementary Fig. 14), which possibly reduced its reaction rate with sulfide and phosphate ions in the wastewater and sludge.

In contrast, E-FeCO_3_, FeCl_2_, and FeCl_3_ were all effective in eliminating sulfide and phosphate and in enhancing sludge settleability and dewaterability (Fig. 5). Specifically, in anaerobic sewage, the dissolved sulfide control efficiencies of E-FeCO_3_, FeCl_2_, and FeCl_3_ were 0.53 ± 0.02 g S/g Fe, 0.51 ± 0.02 g S/g Fe, and 0.56 ± 0.03 g S/g Fe, respectively (Fig. 5a). Dissolved sulfide concentrations below 0.1 mg S/L were achieved in the overdosing tests with all these iron salts (Supplementary Fig. 15a). Similarly, in the anaerobic sludge digesters, all three iron salts reduced the dissolved sulfide and gaseous H_2_S concentrations to below 2 mg S/L and 200 ppmv, respectively, with nearly 90% reduction. (Fig. 5f). The methane production and sulfate reduction processes were not impacted (Supplementary Fig. 15e, f). E-FeCO_3_, FeCl_2_, and FeCl_3_ also displayed similar efficiencies in phosphate removal from aerated sludge, at 0.47 ± 0.02 g S/g Fe, 0.51 ± 0.02 g S/g Fe, and 0.52 ± 0.02 g S/g Fe, respectively (Fig. 5c). Furthermore, the uses of E-FeCO_3_, FeCl_2_, and FeCl_3_ increased the sludge settleability by 36.9 ± 6.2%, 37.4 ± 3.4%, and 49.7 ± 3.6%, respectively, and enhanced the sludge dewaterability by 55.0 ± 1.5%, 57.9 ± 5.3%, and 63.8 ± 3.2%, respectively (Fig. 5h). Overall, the performance of E-FeCO_3_ and FeCl_2_ in sulfide and phosphate removal and in improving sludge settleability and dewaterability is similar, which is also similar to, or slightly lower than that of FeCl_3_. Fe^3+^ is able to oxidize sulfide (in addition to Fe^2+^ and sulfide precipitation) and is also known to have stronger flocculating or coagulating capabilities compared to Fe^2+^, which may explain the performance difference observed. The performance of FeCl_2_ and FeCl_3_ observed in these tests is comparable to that reported in literature (Supplementary Table 2), supporting the reliability of the results reported herein.

In contrast, the doses of E-FeCO_3_, FeCl_2_, and FeCl_3_ induced different pH shifts. Dosages of ~30 mg Fe/L of E-FeCO_3_, FeCl_2_ and FeCl_3_ to anaerobic sewage altered sewage pH by 0.15, −0.02, and −0.13 units, respectively (Fig. 5b and Supplementary Fig. 15b). A higher dose at ~90 mg Fe/L caused pH variations of 0.31, −0.49, and −0.66 units, respectively (Fig. 5b and Supplementary Fig. 15b). Similar pH variation patterns were also observed in the experiments with activated sludge (Fig. 5d and Supplementary Fig. 15d) and anaerobic sludge digesters (Fig. 5g). In all these cases, the provision of additional alkalinity via E-FeCO_3_ addition, in comparison to the consumption of alkalinity via FeCl_2_ or FeCl_3_ dosage, is favorable, as will be further discussed later (Discussion Section).

In conclusion, E-FeCO_3_ proves to be a suitable replacement for FeCl_2_ and FeCl_3_ for wastewater or wastewater sludge management, while C-FeCO_3_ shows limited effectiveness.

### An integrated urban water management strategy

**Fig. 6:**
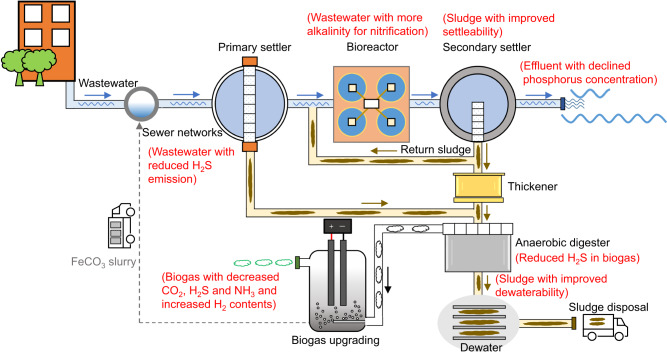
Schematic drawing of our urban wastewater management system. The system includes biogas upgrading and E-FeCO_3_ production and application.

The experimental findings support an integrated urban water management strategy, comprising the production of E-FeCO_3_ at a WWTP via biogas upgrading, and the dosing of E-FeCO_3_ to the upstream sewer catchment for corrosion and odor mitigation with various beneficial flowing-on effects, and/or to various units in the WWTP to achieve phosphorous removal from wastewater, sulfide removal in the anaerobic sludge digester, and to improve sludge settleability and dewaterability (Fig. 6).

The wastewater biodegradable chemical oxygen demand (bCOD) concentration affects the amount of biogas produced, which subsequently determines the amount of E-FeCO_3_ that can be produced. Mass balance analysis shows that the amount of E-FeCO_3_ that can be produced via biogas upgrading can meet the demand for iron salts for these purposes in the same catchment (Supplementary Table 3). Even for sewage with a moderate bCOD concentration of 300 mg/L and a moderate wastewater bCOD to methane conversion ratio of 7%, the iron salts produced would add 12 mg Fe/L of sewage (Supplementary Table 3), adequate for all the above-mentioned purposes^10, 14^.

A full economic analysis of the proposed process is not possible before the process is scaled up. An input-output analysis is performed for a hypothetical catchment and WWTP with a sewage flow rate of 120 ML/d (Supplementary Table 2). The plant produces biogas at 1641 m^3^/d assuming the influent contains 300 mg/L of bCOD, the upgrading of which produces E-FeCO_3_ at 1470 kg Fe/d and upgraded biogas at 1,641 m^3^/d. Replacing FeCl_2_ for sewer dosing and gasoline as a car fuel, respectively, these products entail a combined output value of A$2.1 m/y. In comparison, the combined costs for the input materials (biogas, electricity, NaCl, and recycled iron) are estimated to be A$0.64 m/y.

The life-cycle environmental impacts of the proposed process (Scenario A), with E-FeCO_3_ replacing FeCl_2_ for in-sewer dosing and the upgraded biogas as a car fuel, were compared with the status quo (Scenario B), with FeCl_2_ produced from steel pickling and biogas used for combined heat and electricity production (Supplementary Fig. 16). Scenario A is further divided into A1 and A2 with the electricity for E-FeCO_3_ production generated from biogas (A1) and from the current mix of primary energy sources in Australia (A2), respectively. Scenario B is also divided into B1 and B2 with FeCl_2_ transported for 1000 km and 4000 km, respectively.

**Fig. 7:**
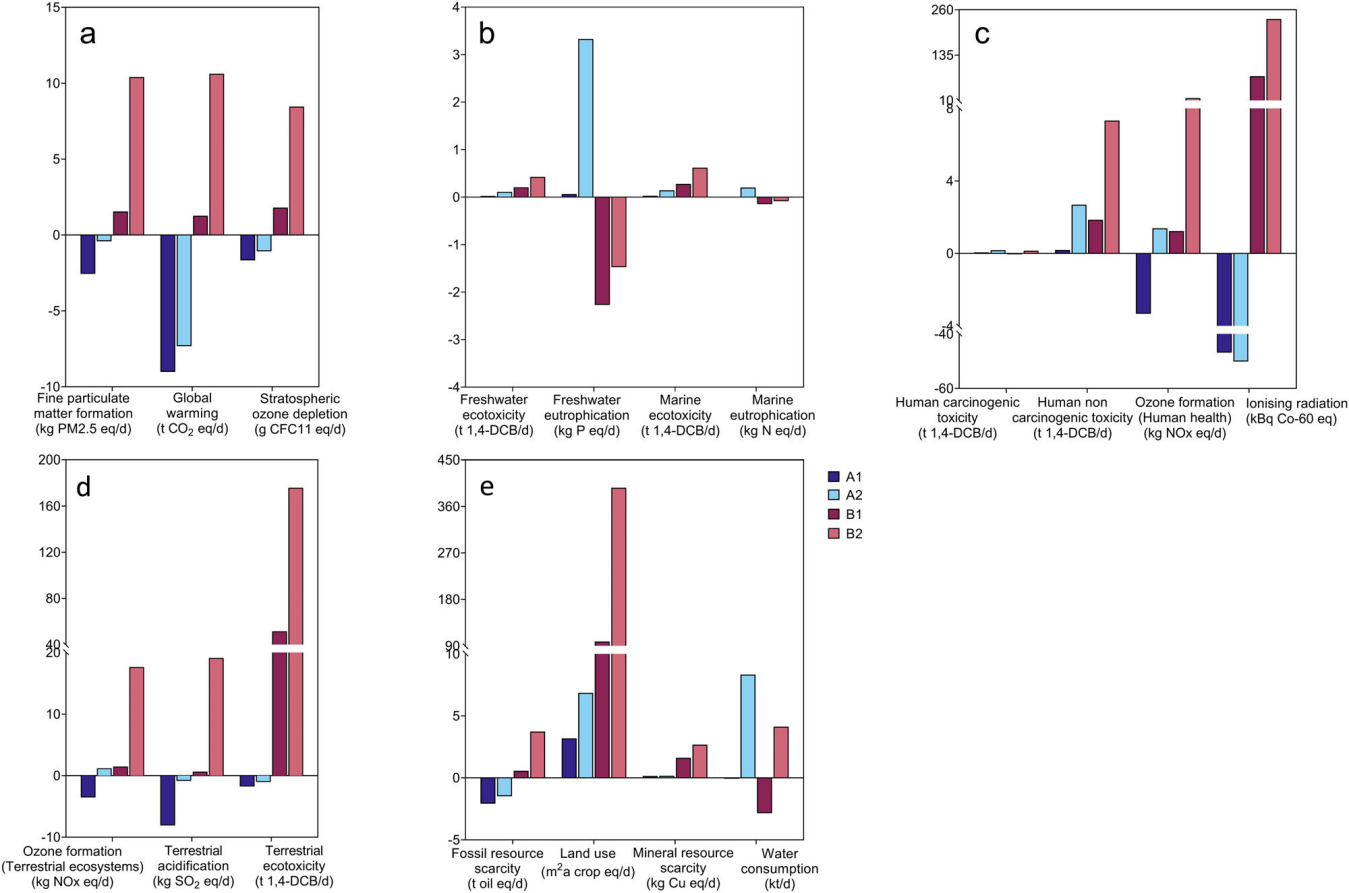
Life cycle assessments of four different iron supply scenarios. **a** Atmospheric environment impacts. **b** Aqueous environment impacts. **c** Human health impacts. **d** Terrestrial environment impacts. **e** Resource usage impacts.

The status quo Scenarios B1 and B2 have negative environmental impacts against almost all indicators (Fig. 7), as the environmental impacts of FeCl_2_ production and transportation could not be completely offset by the combined heat and electricity production from biogas, with indicators of Freshwater Eutrophication and Marine Eutrophication being two exceptions (Supplementary Fig. 17).

In contrast, Scenario A1 delivers positive or negligible environmental impacts in all categories (Fig. 7), owing to (1) the positive environmental impacts achieved with the replacement of gasoline with the upgraded biogas as a car fuel, and (2) the negligible or even positive (via CO_2_ fixation) environmental impacts of E-FeCO_3_ production. Consequently, A1 substantially outperforms B1 and B2 in most categories.

Different from A1, grid electricity is used to drive the electrochemical cell in A2. The current energy mix for power production in Australia has coal as a key component (54.9%). The impacts of coal use against several indicators could not be completely offset by the substitution of gasoline with upgraded biogas (Supplementary Fig. 17), due to the much lower impacts of gasoline on these indicators than coal. Nevertheless, A2 outperforms B1 and B2 against 13 of the 18 indicators. With the continued shift towards renewables in the energy mix, the environmental performance of A2 is expected to further improve in the years to come.

## Discussion

We are entering an era of circular economy. This requires us to improve our traditional approaches to cater to the requirement of sustainable development. This study showcases an integrated technological solution within an urban water system. It establishes a solid connection between biogas upgrading and the enhancement of wastewater and sludge management. Specifically, it offers the potential to protect sewer infrastructure, facilitate the removal and recovery of nutrients from wastewater, and reduce costs associated with sludge disposal. The experimental findings demonstrated the feasibility of this out-of-the-box solution, highlighting its ability to address multiple challenges simultaneously.

An electrochemical cell was implemented to achieve the multiple goals. The construction of this electrochemical cell is straightforward, utilizing iron plates as electrodes and a NaCl solution as the electrolyte, without requiring complex materials or membranes. The iron should ideally be sourced from locally recycled iron. If this is not available, it could be imported from iron manufactures. Even in the latter case, the transport costs would be greatly reduced. As an example, the FeCl_2_ supplied in Australia is a solution containing 12% iron. Theoretically, transporting iron as iron plates instead of this iron salt solution would substantially reduce the transportation cost, with a ~88% reduction in weight and a ~98% reduction in volume. In addition, this electrochemical cell can be integrated into existing wastewater treatment systems, following the AD process (Fig. 6). Furthermore, the system is easy to operate, and the control logic is simple. By adjusting the current to keep the electrolyte pH at a pre-selected level (recommended to be around 8.5), the amount of OH^−^ produced is ensured to just meet the demand for CO_2_ conversion to CO_3_^2−^ and its subsequent removal as FeCO_3_.

Serving as scarifying electrodes, the Fe plates need to be replaced regularly, with an interval determined by the reactor size, the plate spacing, and the CO_2_ loading rate. In the example used in the input-output analysis, the CO_2_ loading rate is estimated to be in the range of 656−1750 m^3^/d, given a bCOD concentration range of 300−800 mg/L (Supplementary Table 4). Assuming a total electrochemical cell volume of 200 m^3^ and an iron plate spacing of 1 cm, the Fe plate replacement interval is estimated to be 4−10 months (Supplementary Table 4), which is reasonable.

The overall process comprises three key steps, namely the electrochemical production of Fe^2+^ and OH^−^, dissolution of CO_2_ and its subsequent conversion to CO_3_^2−^ under alkaline conditions, and the precipitation of Fe^2+^ and CO_3_^2-^ as FeCO_3_. Among these, the CO_2_ transfer from the gas bubbles to the electrolyte is the rate-limiting step, which determines the rate of the overall electrochemical system. The CO_2_ mass transfer rate is influenced by the reactor configuration, the gas flow rate, the gas bubble size, and the operating pH. In our proof-of-concept experiments at an optimal pH of 8.5, ~85% of CO_2_ in the feed gas was retained in the reactor as FeCO_3_, while the remaining ~15% remained in the upgraded gas. The CO_2_ removal efficiency can be further improved through reactor engineering and gas flow rate control (Fig. 2c). CO_2_ removed from the feed gas was replaced by H_2_ generated at the cathode with a molar ratio of 1:1. The energy content of H_2_ in the upgraded biogas partially recovers the electricity energy invested.

This study proposed and experimentally demonstrated, that E-FeCO_3_, despite in a solid form as particles, can replace soluble iron salts for wastewater management. However, the industry survey conducted in Australia and a more recent comprehensive literature review both showed the C-FeCO_3_ had not been applied to wastewater and sludge management^7,36^. Our comparative experiments showed that the C-FeCO_3_ is ineffective in sulfide or phosphate precipitation (Fig. 5), due to its stable crystalised structure in larger particles (Supplementary Fig. 14). The low reactivity of C-FeCO_3_ limits its applications in urban water management.

Compared to FeCl_2_ and FeCl_3_, the dosage of E-FeCO_3_ to wastewater or sludge causes a slight rise rather than a drop of pH, as in the case of FeCl_2_ and FeCl_3_ dosing. This is because the dosage of E-FeCO_3_ provides additional alkalinity. In comparison, the dosage of FeCl_2_ and FeCl_3_ consumes alkalinity. In sewer networks, an increase of sewage pH is desirable as it shifts the H_2_S and HS^−^ equilibrium towards HS^−^ (${{{{{{\rm{H}}}}}}}_{2}{{{{{\rm{S}}}}}}\leftrightarrow {{{{{{\rm{HS}}}}}}}^{-}+{{{{{{\rm{H}}}}}}}^{+}$), and hence reduces the transfer of H_2_S from the liquid to the gas phase. In fact, alkali in the form of e.g. Mg(OH)_2_ is often added to sewage in areas where H_2_S is problematic^7,36^, clearly illustrating the importance of pH elevation for H_2_S control in sewers. In the wastewater treatment process, the additional alkalinity provided via E-FeCO_3_ dosage is potentially beneficial for nitrification, particularly for wastewaters containing relatively low levels of alkalinity. Although denitrification partially regenerates alkalinity consumed by nitrification, wastewater in some parts of the world still does not contain alkalinity at a level enabling satisfactory nitrogen removal^37,38^. In such cases, E-FeCO_3_ should be a better source of iron than FeCl_2_ or FeCl_3_. Also, pH stability is critical for anaerobic sludge digesters. The additional alkalinity provided with E-FeCO_3_ dosage helps improve pH stability in the digesters.

Fe^3+^ salts are sometimes dosed to the primary settling tank in a wastewater treatment plant, or to the secondary effluent to remove phosphate. These cannot be replaced with Fe^2+^-based salts including E-FeCO_3_. However, E-FeCO_3_ (indeed any other Fe^2+^-based salts as well) could be added to the aeration basin to remove phosphate (Fig. 2).

The majority of iron dosed to the wastewater or the wastewater sludge will end in the biosolids as solid iron salts including iron-phosphate compounds, e.g. Fe_3_(PO_4_)_2_ (vivianite)^39^, which increases sludge production. However, as demonstrated in this as well as previous work, the addition of iron salts helps improve sludge settleability and dewaterability, which means additional sludge production may not necessarily lead to increased sludge disposal costs.

Numerous engineering aspects require further research in the upscaling of the process. In this proof-of-concept study, biogas was distributed to a short reactor using a 0.5 mm diameter needle. In full-scale applications, we expect that the biogas will be provided via a gas distribution system generating microbubbles in a relatively tall reactor. The reactor should be designed such that an adequate gas retention time is achieved for CO_2_ in the up-traveling gas bubbles to diffuse into the liquid phase and dissociate as bicarbonate and carbonate, before the gas bubbles reach the reactor headspace releasing CH_4_ and H_2_. The electrode should be designed such that Fe^2+^ can be produced at a rate required for CO_2_ removal with an acceptable voltage and power consumption. Also, experiments should be performed at full-scale sewer networks, wastewater treatment plants, and anaerobic sludge digesters, operated in a variety of conditions, to test the effectiveness of the E-FeCO_3_ slurry for the intended purposes.

## Methods

Numerous experiments were conducted to demonstrate the proposed concepts. The overall structure of the experimental design is shown in Supplementary Fig. 18, with details of each experiment described below.

### Electrochemical cell setup and operation

The CO_2_ removal tests were conducted in a modified glass bottle with a total volume of 325 mL in a fume hood in a temperature-controlled (22 ± 1 °C) laboratory (Supplementary Fig. 1). The reactor was sealed to ensure gas-tightness and mixed with a magnetic stirrer at a speed of 300 rpm. Two iron plates (mild steel, Harding Steel), served as anode and cathode, respectively, were placed in parallel and fixed to the lid of the bottle, with an interelectrode gap of 1.0 cm. The dimensions of the iron plates were 15 cm × 1.4 cm × 0.3 cm. Each iron plate was submerged at a depth of 3.5 cm in the electrolyte, achieving a submerged surface area of 11.9 cm^2^. Iron oxidation was achieved by controlling the electrochemical cell current via a bench power supply (72-2685, TENMA, China). The feed gas was diffused into the electrolyte via a 0.5 mm diameter needle. Due to safety concerns, the feeding gas in all but one test comprised ~60% N_2_ and ~40% CO_2_ with N_2_ as a proxy of CH_4_ as both have a low solubility. In one test, the feed gas comprised ~60% CH_4_ and ~40% CO_2_, for comparison with results from tests with N_2_, as well as trace levels of H_2_S (884.1 ± 63.5 ppmv) and NH_3_ (267.5 ± 26.1 ppmv) to evaluate the capability of the electrochemical cell to remove these contaminants. The feeding gas flow was controlled with a gas flow controller (Bronkhorst, Netherlands), with the ‘upgraded gas’ collected with a 5 L gas bag connected to the reactor outlet. In each test, 200 mL of 2 g/L of NaCl solution, prepared using tap water, was used as the electrolyte after being sparged with the feed gas for 30 min at a flow rate of 0.1 L/min, to remove the residual dissolved oxygen (DO). pH in the reactor was monitored with a portable pH meter (miniCHEM, Labtek). The reactor has sampling ports for gas, liquid, and solids sampling, as shown in Supplementary Fig. 1.

### Electrochemical CO_2_ removal and E-FeCO_3_ production

The CO_2_ removal efficiency of the cell was evaluated in triplicate at pH 7.5, 8.0, 8.5 and 9.0, respectively, via a series of batch tests. The tests at pH 8.5 were repeated with gas feed composition changed from N_2_ (~60%) and CO_2_ (~40%) to CH_4_ (~60%), CO_2_ (~40%), H_2_S (884.1 ± 63.5 ppmv) and NH_3_ (267.5 ± 26.1 ppmv). Each test lasted for 6 h, comprising 2 h of preparatory phase, and 4 h of experimental phase. Initially, 200 mL of oxygen-free electrolyte was added into the reactor, leaving 125 mL as the headspace. In the preparatory phase, a current was supplied to the cell in the absence of a gas supply. pH in the reactor was progressively elevated to the pre-specified level (i.e. 7.5, 8.0, 8.5 or 9.0) due to the on-going production of hydroxide (along with H_2_) in the cell. The subsequent experimental phase commences when the pH set-point was reached, during which the feed gas was fed into the reactor at a rate of 5 mL/min. The current in the experimental phase was further manually adjusted so that the pH was maintained at the set-point (i.e. 7.5, 8.0, 8.5 or 9.0). This adjustment was only needed at the beginning of the phase, as pH remained stable once a suitable current was found, due to the following reactions:$\,{{{{{\rm{Fe}}}}}}+2{{{\mbox{H}}}}_{2}{{\mbox{O}}}\to {{{{{{\rm{Fe}}}}}}}^{2+}+{{2{{\mbox{OH}}}}^{-}+{{{{{\rm{H}}}}}}}_{2}$ and ${{{{{{\rm{CO}}}}}}}_{2}+{{{{{{\rm{H}}}}}}}_{2}{{{{{\rm{O}}}}}}+{{{{{{\rm{Fe}}}}}}}^{2+}\to {{{{{{\rm{FeCO}}}}}}}_{3}+2{{{{{{\rm{H}}}}}}}^{+}$.

Gas samples were taken from the headspace of reactor with a 100 μL syringe hourly in the first 4 h, and then every half hour in the last 2 h. The liquid and solid samples were taken hourly for the analysis of iron concentration. The voltage was recorded every 5 min manually. About 50 mL Fe-containing slurry freshly produced at pH 8.5 was collected for XRD analysis, a further 3 mL sample was collected for particle size distribution analysis. Finally, at the end of each test, all the liquid and solid content in the reactor was transferred into a 200 mL oxygen-free sealed bottle for further experiments as described below.

One additional set of experiments was conducted at pH 8.5, aimed to evaluate the effect of gas flow rate on the cell performance. The test lasted for 9 h, comprising a 2 h preparatory phase with pH elevated to 8.5 in the absence of a gas supply, and a 7 h experimental phase, during which the gas flow rate was stepwise increased from 2 mL/min (3 h) to 5 mL/min (2 h), and further to 10 mL/min (2 h). The current was manually adjusted following each change of the gas flow to ensure a constant pH at 8.5. Gas samples were taken hourly in the first 4 h, and then every half hour in the following 5 h.

### E-FeCO_3_ as an iron salt to support urban wastewater management

Two sets of experiments were designed to assess the suitability of E-FeCO_3_ produced in biogas upgrading for supporting urban wastewater management. The first set was designed to assess the effects of E-FeCO_3_ slurry dosing to sewers on sulfide control, to a biological wastewater treatment reactor on phosphate removal, and to an anaerobic sludge digester on sulfide control. In the second set, the flow-on effects of in-sewer dosed E-FeCO_3_ slurry on the performances of biological wastewater treatment system and anaerobic digestion were investigated, noting that an urban wastewater system is an integrated system.

Wastewater was collected from a local domestic wastewater pump station (Brisbane, Australia), and stored at 4 °C prior to use to minimize changes in wastewater characteristics. It had a pH of 7.1–7.4 and contained total COD at 400–600 mg/L including soluble COD at 220–310 mg/L, phosphate at 4−7 mg P/L, iron at 0.1–0.3 mg Fe/L, sulfate at 10–20 mg S/L, sulfide at 5–10 mg S/L, and undetectable levels of oxygen. Activated sludge was collected from a local WWTP (Brisbane, Australia), with a mixed liquor suspended solids (MLSS) and a mixed liquor volatile suspended solid (MLVSS) concentration of 13.2 ± 0.1 g/L and 10.6 ± 0.1 g/L, respectively. Anaerobically digested sludge was collected from a laboratory anaerobic digestion reactor, with the total solid (TS) and volatile solid (VS) concentrations of 20.6 ± 0.1 g/L and 16.3 ± 0.1 g/L, respectively.

The E-FeCO_3_ slurry produced at pH 8.5 was used to conduct all these experiments.

#### The effect of E-FeCO_3_ slurry on sulfide control in sewer

For each sulfide removal experiment in sewer, wastewater of 290 mL was filtered using disposable millipore filter units (0.45 *μ*m), and then transferred into a 300 mL sealed bottle. The bottle was stripped with pure nitrogen gas for 30 min to further remove dissolved oxygen. A sulfide stock solution (Na_2_S·9H_2_O of ~1.5 g S/L) of 5 mL was then added to the bottle to increase the sulfide concentration to ~25 mg S/L, followed by the addition of 1 M HCl to obtain a pH of 7.2, typical of domestic wastewater. After that, a pre-determined amount of the E-FeCO_3_ slurry was added to each experiment to achieve a pre-designed initial iron concentration (described below). To guarantee there was no headspace during the experiment, two syringes filled with filtered and oxygen-free wastewater, were connected to the reactor to replenish the reactor after sampling. Each test lasted for 3 h, during which the reactor was mixed with a magnetic stirrer at 300 rpm. Liquid samples were taken before E-FeCO_3_ dosing, and every 15 min in the first hour after the dosing, and then every 30 min, for the measurement of dissolved sulfide. pH in the reactor was monitored with a portable pH meter and recorded manually at the same intervals. An additional sample was taken at the end of each test to measure the total iron concentration.

Two different initial Fe levels, namely 30 and 90 mg Fe/L, were used in the above-described experiments. According to the theoretical reaction stoichiometry (${{{{{{\rm{Fe}}}}}}}^{2+}+{{{{{{\rm{S}}}}}}}^{2-}\to {{{{{\rm{F}}}}}}{{{{{\rm{e}}}}}}{{{{{\rm{S}}}}}}\downarrow$), an initial Fe concentration of 30 mg/L is insufficient for removing the sulfide initially present in the wastewater (~25 mg S/L), and hence the ratio between sulfide removed and Fe added could be determined. In contrast, Fe would be in excess for an initial Fe concentration of 90 mg Fe/L, and hence the lowest achievable sulfide concentration can be determined.

The above experiments were performed with both freshly produced E-FeCO_3_ slurry i.e. with experiments undertaken within 1 day following the E-FeCO_3_ production, and E-FeCO_3_ slurry stored in a sealed serum bottle at a temperature-controlled (22 ± 1°C) laboratory for 1, 2 and 4 weeks to determine the impact of E-FeCO_3_ storage on the sulfide removal performance.

#### Suspension of E-FeCO_3_ particles in sewer

The electrochemically produced E-FeCO_3_ was in a slurry. For its use in sewers for sulfide control, it should remain in suspension after addition to sewage under in-sewer hydrodynamic conditions. Batch tests were therefore conducted in a 200 mL reactor that was mixed with a magnetic stirrer at an intensity that creates turbulence, as described by the Reynolds number, similar to that in gravity or rising main sewers. At the start, 198 mL of tap water, stripped with nitrogen gas for 30 min to remove the DO, was transferred to the bottle, followed by the injection of 2 mL E-FeCO_3_ slurry with a syringe. The iron concentration thus obtained is estimated to be ~100 mg Fe/L, simulating an overdosing situation. Each test lasted for 30 min. Liquid samples were taken through the middle sampling port, immediately after the E-FeCO_3_ dosing and at the end of the test, for the measurement of total iron concentration. Identical iron concentrations would indicate the absence of E-FeCO_3_ settling.

The experiments were performed with both freshly produced E-FeCO_3_ slurry and E-FeCO_3_ slurry stored for 1, 2, and 4 weeks to determine the impact of E-FeCO_3_ storage on the sulfide removal performance. The particle size distributions in the stored slurries were measured prior to use.

#### The effect of E-FeCO_3_ slurry on phosphate removal during wastewater treatment

For each phosphate removal test, activated sludge of 100 mL was mixed with 400 mL filtered wastewater, with the mixture transferred to a 1 L bottle. A phosphate stock solution (5 g P/L of KH_2_PO_4_) of 1.5 mL was then added to the bottle to increase the phosphate concentration to about 20 mg P/L. The E-FeCO_3_ slurry (∼10 g Fe/L) of about ∼0.8 and ∼3.5 mL was dosed to different experimental bottles to obtain two levels of initial Fe concentrations, namely ∼16 and ∼70 mg Fe/L. Control tests were also conducted without iron dosing. Each test lasted for 6 h, during which the DO concentration was controlled at 2.0–3.0 mg O_2_/L with a programmable logic controller (PLC) via on/off control of the air flow. The reactor was mixed with a magnetic stirrer at 300 rpm. Liquid samples were taken before E-FeCO_3_ dosing, and every 0.5 h in the initial 2 h, and then hourly, for the measurement of phosphate concentration. The reactor pH was monitored with a portable pH meter and recorded manually with the same intervals. An additional sample was also taken at the end of each test for the measurement of the total iron concentration.

#### The effect of E-FeCO_3_ slurry on sulfide control in anaerobic digestion

The effect of E-FeCO_3_ on sulfide control in anaerobic digestion was evaluated via biochemical methane potential (BMP) tests, conducted according to the standard procedure^40^. Specifically, about 20 mL thickened activated sludge (TS: 21.3 ± 0.1 g/L; VS: 17.7 ± 0.1 g/L) was mixed with ∼40 mL inoculated digested sludge (TS: 20.6 ± 0.1 g/L; VS: 16.3 ± 0.1 g/L), and then transferred into a 100 mL sealed bottle. A blank test was also carried out using the feeding of ~20 mL wastewater and ∼40 mL inoculated digested sludge. After that, the sealed bottle was stripped with pure nitrogen gas for 10 min to remove the residual oxygen. The E-FeCO_3_ slurry (∼10 g Fe/L) of about ∼0.8 mL was dosed to the experimental bottles to obtain an initial Fe concentrations of ~80 mg Fe/L. After that, a sulfate stock solution (Na_2_SO_4_ of ~1.5 g S/L) of 1.0 mL was added into the reactor to increase the sulfate concentration to about 25 mg S/L, followed by the addition of 1 M HCl to adjust the reactor pH to ~7.5, typical for anaerobic sludge digester. Afterwards, all the BMP bottles were incubated in a temperature-controlled (37 ± 1 °C) incubator. The BMP tests lasted for about 30 days until almost no further increase of biogas was detected. Gas samples were taken every 2 days in the initial 10 days, and every 5 days to the end, for the measurement of the content of N_2_, CH_4_, and CO_2_ in the biogas. The volume of biogas produced in each BMP bottle was also measured at the same intervals. The gas pressure in each BMP bottle was regularly assessed using a manometer (Testo, Australia) prior to each sampling event. The volume of newly generated biogas was determined by calculating the difference in gas pressure between two consecutive sampling events. Gas and liquid samples were taken every 5 days for the measurement of inorganic sulfur species. An additional sludge sample was also taken at the end of each test for measuring sludge dewaterability.

#### The flow-on effects of in-sewer dosed E-FeCO_3_ slurry on downstream wastewater and sludge treatment

The effect of in-sewer dosed E-FeCO_3_ slurry on the biological wastewater treatment process was investigated in two steps, namely sulfide removal in sewer followed by phosphate removal during aerobic treatment of the E-FeCO_3_-receiving wastewater with activated sludge. The sulfide removal step was performed as per the previous description, with freshly produced E-FeCO_3_ slurry. The initial sulfide and Fe concentrations in this test were ~18 mg S/L and ∼20 mg Fe/L, respectively, ensuring that E-FeCO_3_ was not in excess. After the 3 h sulfide removal test, the 300 mL E-FeCO_3_-dosed sewage was fed to 300 mL activated sludge which was prepared by mixing 150 mL activated sludge with the raw wastewater at a ratio of 1:1 (v/v). A phosphate stock solution (5 g P/L of KH_2_PO_4_) of 3.0 mL was then added to the bottle to increase the phosphate concentration to about 25 mg P/L. Each test, with the mixed liquor aerated, lasted for 6 h, with the operational and monitoring procedures identical to those applied in the above-described phosphate removal test.

The effect of in-sewer dosed E-FeCO_3_ slurry on the anaerobic sludge digestion was investigated in three steps, sulfide removal in sewer, phosphate removal during wastewater treatment, and sulfide control in anaerobic digestion. The first two steps were similar to the above-described experiments investigating the flow-on effect on phosphate removal, with the following differences. The initial sulfide, Fe, and phosphate concentrations in this experiment were much higher than those in the above-described experiments, being about 200 mg S/L, 300 mg Fe/L, and 300 mg P/L, respectively. This is because the in-sewer dosed Fe would accumulate in the activated sludge in a practical scenario. It was reported that Fe can accumulate at a concentration 20× that in the wastewater^41^. Following the P-removal test, a sludge sample of 100 mL was harvested for the measurement of sludge settleability, with the remaining sludge centrifuged at 700 × g for 3 min. In the third step, the concentrated sludge (∼15 g VS/L) was used as the feed for BMP tests, to evaluate the effect of in-sewer dosed E-FeCO_3_ on sulfide control in anaerobic sludge digestion. The operational conditions were similar to that mentioned in the Section on The effect of FeCO_3_ slurry on sulfide control in anaerobic digestion.

#### Comparison of E-FeCO_3_ with other iron salts in wastewater and sludge management performance

The performance of E-FeCO_3_, C-FeCO_3_, FeCl_2_, and FeCl_3_ in sulfide and phosphate removal from wastewater/sludge, and in sludge settleability, and dewaterability enhancement were compared via parallel experiments. The four iron salts were separately dosed to anaerobic sewage, aerated activated sludge, and anaerobic sludge digester, respectively. The operational conditions and experimental procedure were as described in the first set of experiments in Section E-FeCO_3_ as an iron salt to support urban wastewater management. The C-FeCO_3_ utilized in this study was procured from Lianyungang Huaihua International Trade Co., LTD. Additionally, the FeCl_2_‧4H_2_O and FeCl_3_‧6H_2_O reagents were acquired from Westlab, Australia.

### Chemical analysis

The detection methods used in study, including MLSS, MLVSS, TS, VS, sludge volume index (SVI), TCOD, SCOD, gaseous CH_4_, CO_2_, and H_2_, total Fe, and specific resistance to filtration (SRF), have been elaborated in Supplementary Table 5. Liquid samples were taken using a syringe and filtered through disposable Millipore filter units (0.22 μm, Millipore, Millex GP) for the analyses of ammonium, nitrite, nitrate, phosphate and inorganic sulfur species (i.e. sulfide, sulfate, silfite and thiosulfate). Ammonium, nitrite, nitrate, and phosphate were analysed using a flow injection analyzer (Lachat Instrument, Milwaukee, Wisconsin), and the sulfur species were measured by Ion Chromatography with an ultraviolet (UV) and conductivity detector (Dionex ICS-2000)^42^. Particle size was measured using dynamic light scattering (Zetasizer Nano ZS, Malvern Instruments). SRF, a common index of sludge dewaterability, was analyzed by using a multi-couple measuring device, as described in literature^43^. The XRD patterns were generated using an X-ray diffractometer (Bruker D8). Prior to XRD measurement, the Fe-containing slurry was dried under vacuum conditions (−50°C, 0.1 mbar), and then ground into powder under anaerobic condition.

### Life cycle assessment (LCA)

The life cycles of two different iron salt supply scenarios for a hypothetical 120 ML/d WWTP were evaluated in this study (Supplementary Fig. 14). Scenario A represents the E-FeCO_3_ approach proposed in this study, including the use of upgraded biogas to replace gasoline as car fuel and the use of E-FeCO_3_ to bring multiple benefits to the wastewater treatment system. Scenario B represents a status quo FeCl_2_ supply approach, including the production and transportation of FeCl_2_ as well as the utilization of biogas for combined power and heat production. Further details of the scenario modeling are provided in Supplementary Table 6 and 7. The impact assessment was carried out using the ReCiPe 2016 Midpoint (H) method in the openLCA 1.10 software. In total, the software estimates 18 environmental impacts. To address the uncertainty of model parameters, 10,000 Monte Carlo simulations were conducted. The detailed uncertainty analysis results are shown in Supplementary Table 8.

### Statistical analysis

To identify the significant difference between experimental and control tests, a student *t*-test was performed in Microsoft Excel. If the *P-*vale is below 0.05, it means the difference is significant, and vice versa.

### Supplementary information

Supplementary Information

### Source data

Source Data

Peer Review File

## Data Availability

The authors declare that the data supporting the findings of this study are available within the paper and its supplementary information files. Source data are provided with this paper.
