# Supplementary material for: Integrated urban water management by coupling iron salt production and application with biogas upgrading
Source: Nat Commun. 2023 Oct 12;14:6405. doi: 10.1038/s41467-023-42158-w (PMC10570337; doi:10.1038/s41467-023-42158-w)
Supplement: Supplementary file 1 — Supplementary Information [file 41467_2023_42158_MOESM1_ESM.pdf]

Supplementary Information

Integrated urban water management by coupling iron salt production and application with biogas upgrading

Zhetai Hu[1], Lanqing Li[2,3], Xiaotong Cen[1], Min Zheng[1], Shihu Hu[1], Xiuheng Wang[2,3], Yarong Song[1], Kangning Xu[4] and Zhiguo Yuan[1,5]*

[1]Australian Centre for Water and Environmental Biotechnology, The University of Queensland, St Lucia, Queensland 4072, Australia. [2]State Key Laboratory of Urban Water Resource and Environment, Harbin Institute of Technology, Harbin, 150090, PR China. [3]School of Environment, Harbin Institute of Technology, Harbin, 150090, PR China. [4]Beijing Key Laboratory for Source Control Technology of Water Pollution, Collage of Environmental Science and Engineering, Beijing Forestry University, Beijing 100083, China. [5]School of Energy and Environment, City University of Hong Kong, Hong Kong SAR, China.

***Corresponding author**

Email address: zhigyuan@cityu.edu.hk (Zhiguo Yuan)

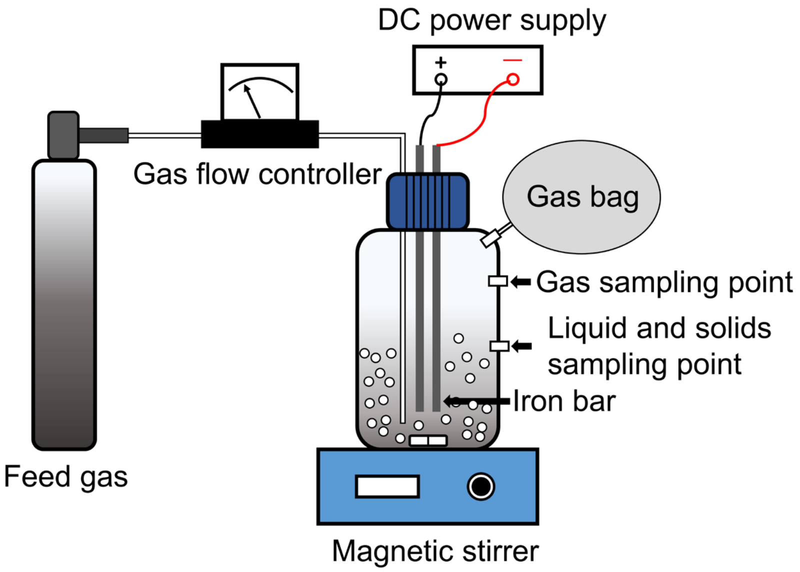

Supplementary Fig. 1. Schematic of the experimental setup.

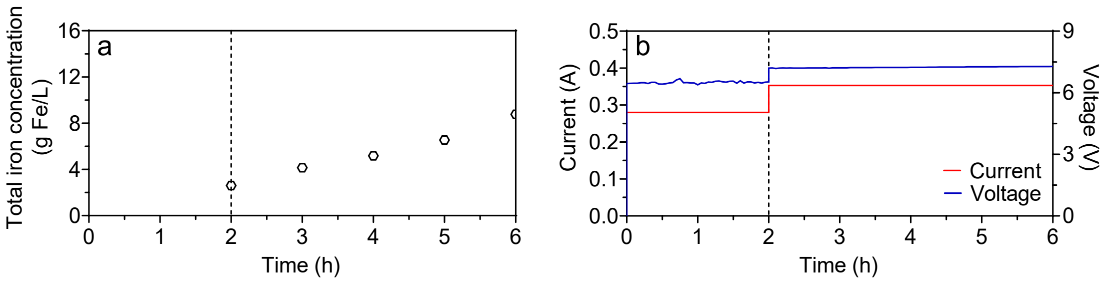

Supplementary Fig. 2. Reactors performance in the tests at pH 8.5 with feed gas containing CH_4, CO_2, H_2S and NH_3. a, Total iron concentration. **b,** Current and voltage. The vertical dotted line represents the start of continuous gas feeding (i.e., the commencement of experimental phase). Averages of triplicate experiments are reported, with error bars representing standard deviations.

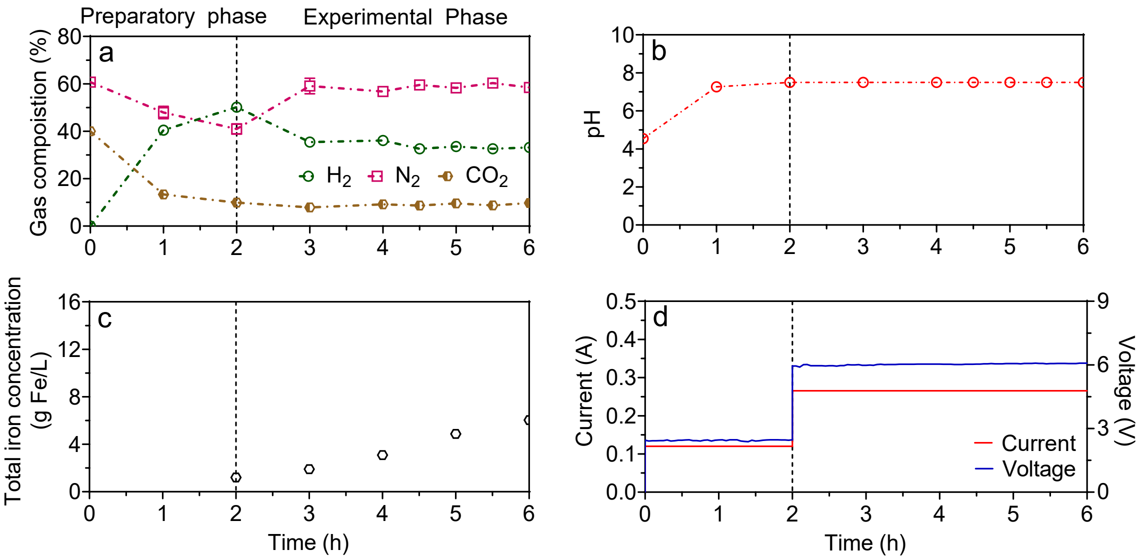

Supplementary Fig. 3. Reactor performance in the tests at pH 7.5. a, H_2, N_2 and CO_2 concentrations in the headspace. **b,** Reactor pH. **c,** Total iron concentration. **d,** Current and voltage applied. The vertical dotted line represents the start of continuous gas feeding (i.e., the commencement of experimental phase). Averages of triplicate experiments are reported, with error bars representing standard deviations.

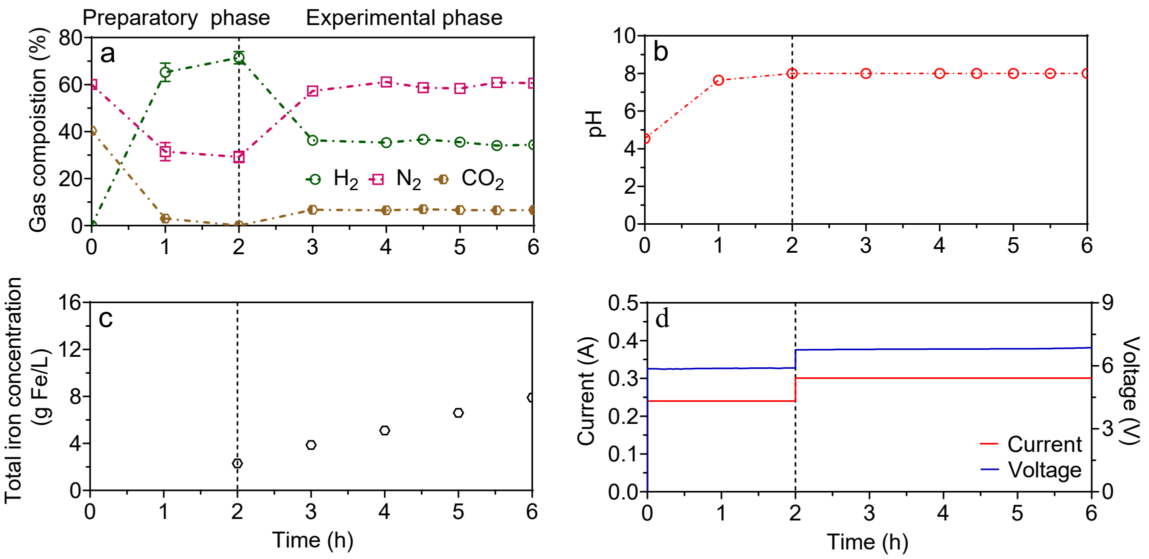

Supplementary Fig. 4. Reactor performance in the tests at pH 8.0. a, H_2, N_2 and CO_2 concentrations in the headspace. **b,** Reactor pH. **c,** Total iron concentration. **d,** Current and voltage applied. The vertical dotted line represents the start of continuous gas feeding (i.e., the commencement of experimental phase). Averages of triplicate experiments are reported, with error bars representing standard deviations.

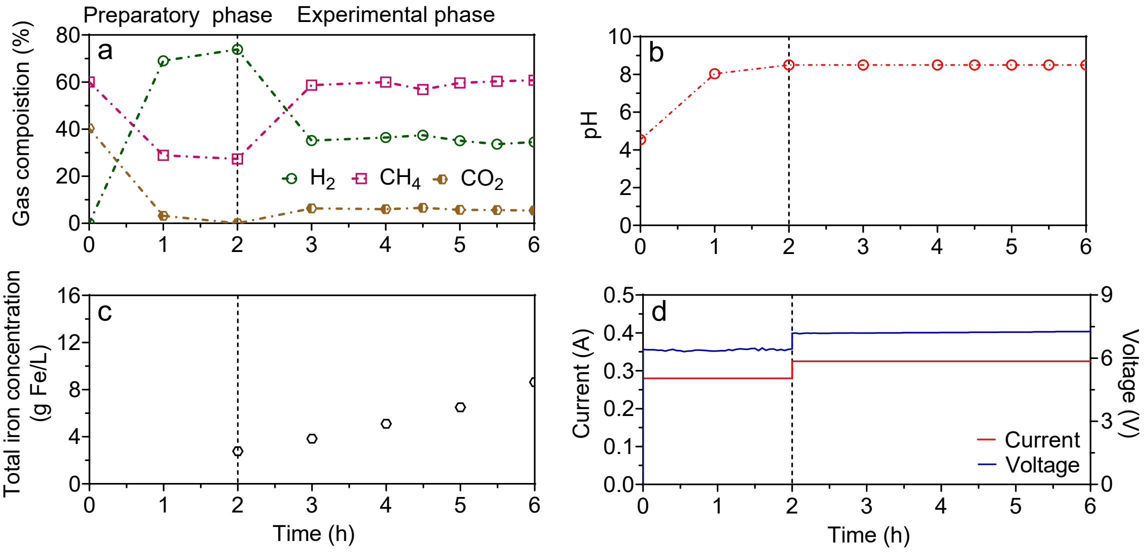

Supplementary Fig. 5. Reactor performance in the tests at pH 8.5. a, H_2, N_2 and CO_2 concentrations in the headspace. **b,** Reactor pH. **c,** Total iron concentration. **d,** Current and voltage applied. The vertical dotted line represents the start of continuous gas feeding (i.e., the commencement of experimental phase). Averages of triplicate experiments are reported, with error bars representing standard deviations.

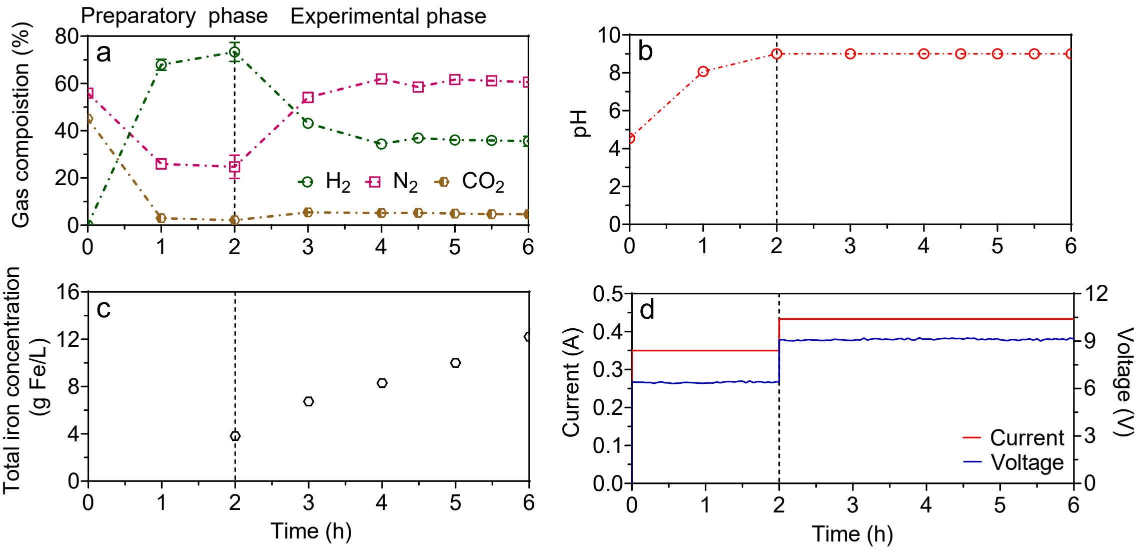

Supplementary Fig. 6. Reactor performance in the tests at pH 9.0. a, H_2, N_2 and CO_2 concentrations in the headspace. **b,** Reactor pH. **c,** Total iron concentration. **d,** Current and voltage applied. The vertical dotted line represents the start of continuous gas feeding (i.e., the commencement of experimental phase). Averages of triplicate experiments are reported, with error bars representing standard deviations.

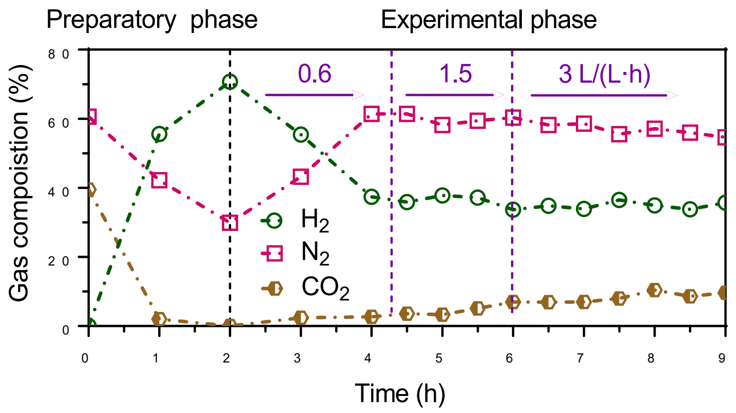

Supplementary Fig. 7. The headspace gas composition in the test with varied gas flow rates (pH = 8.5).

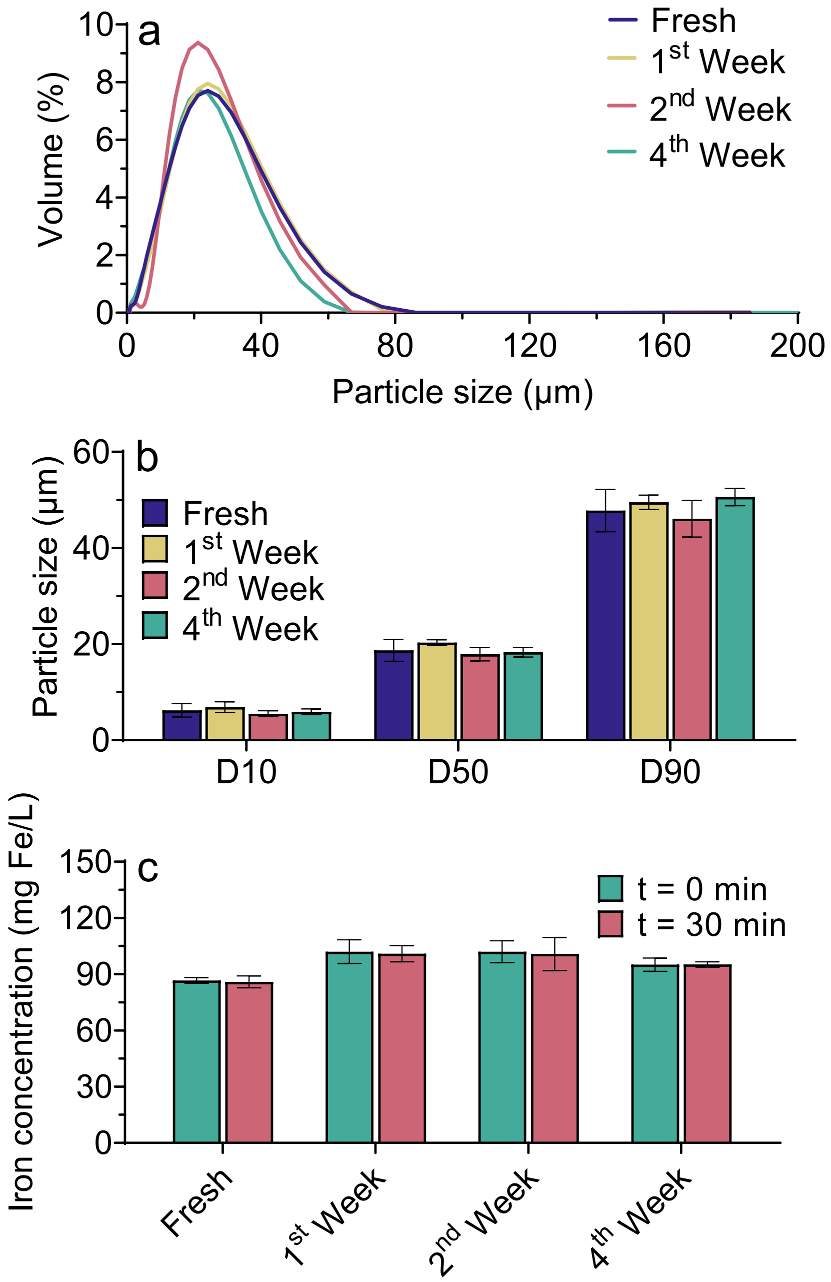

Supplementary Fig. 8. The characteristic of freshly and stored E-$FeCO_3$ particles (produced in experiments at pH 8.5). a, The particle size distribution. **b,** Values of D_{10}, D_{50} and D_{90}. **c,** The suspension performance. All values are means ± standard deviations of triplicate tests.

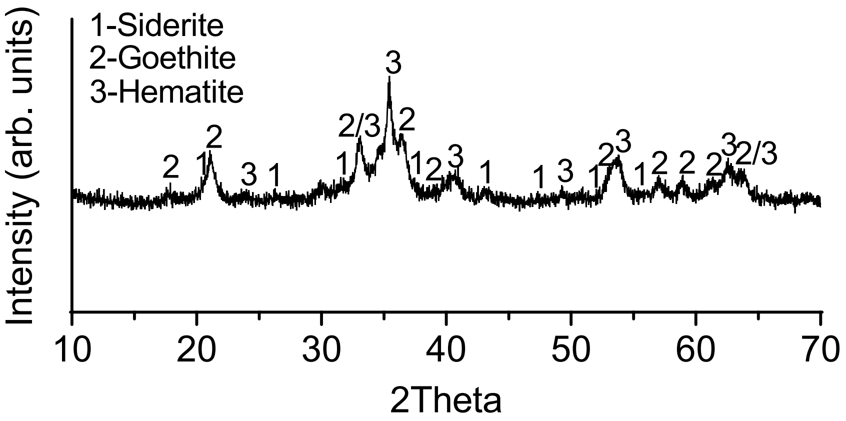

Supplementary Fig. 9. XRD profile of the iron particles produced in tests at pH 8.5.

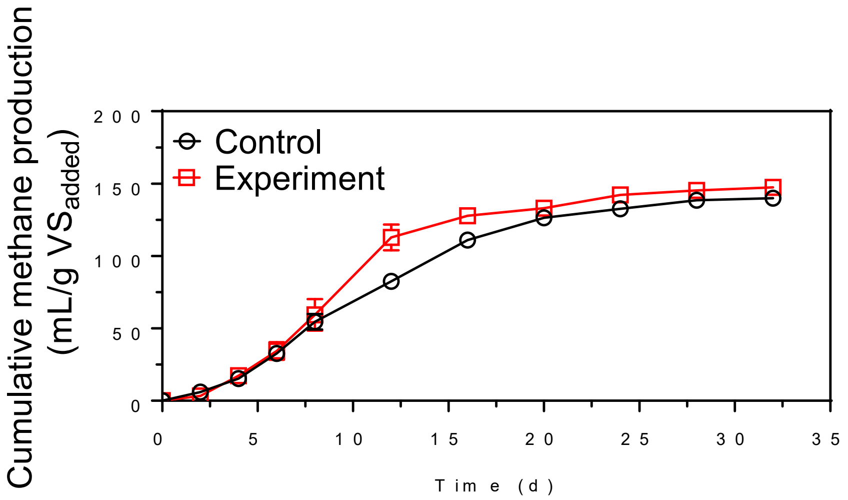

Supplementary Fig. 10. Methane production in the BMP tests with and without E-$FeCO_3$ slurry dosing. Averages of triplicate experiments are reported, with error bars representing standard deviations.

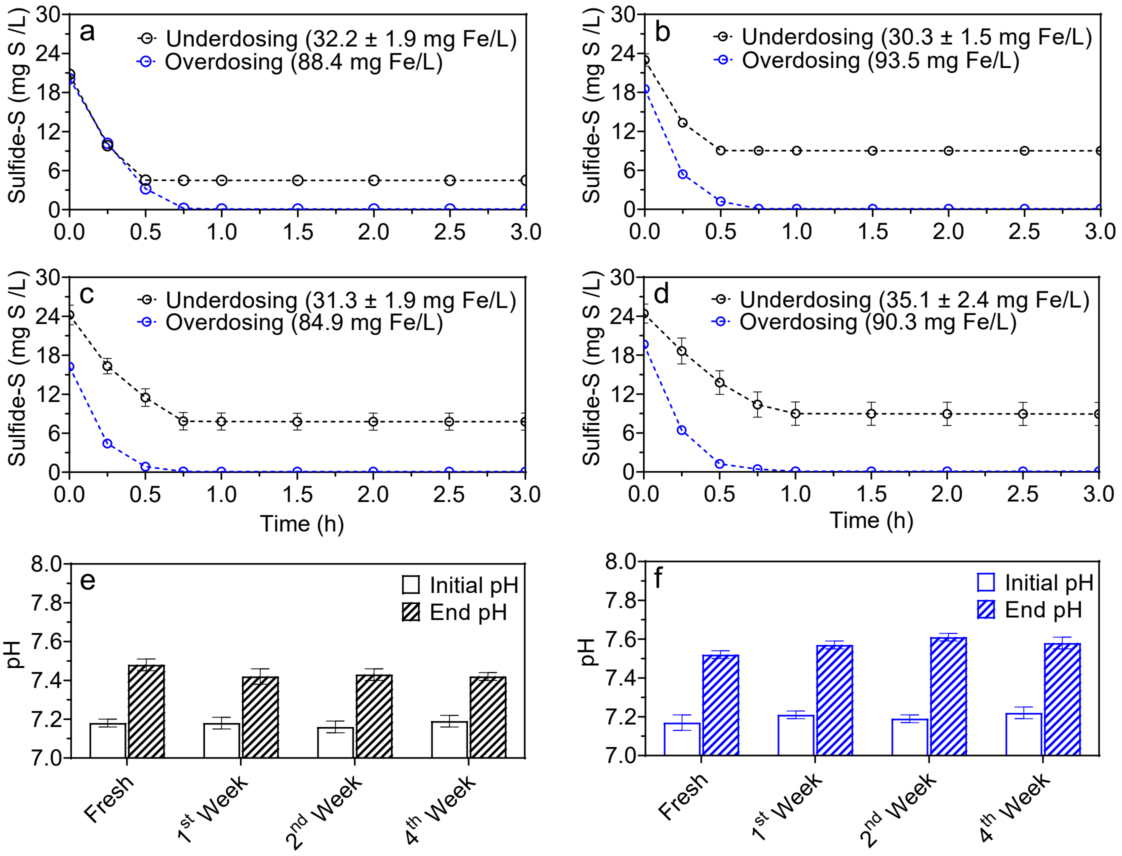

Supplementary Fig. 11. Removal of dissolved sulfide with the E-$FeCO_3$ slurry in sewer condition. The sulfide profiles with dosing of E-$FeCO_3$ slurry **(a)** freshly produced at pH = 8.5, and with dosing of the same E-$FeCO_3$ slurry stored for (**b**) one week, (**c**) two weeks and (**d**) four weeks. pH at the start (prior to dosing) and end of the (**e**) under-dosing and (**f**) overdosing tests. Averages of triplicate experiments are reported, with error bars representing standard deviations.

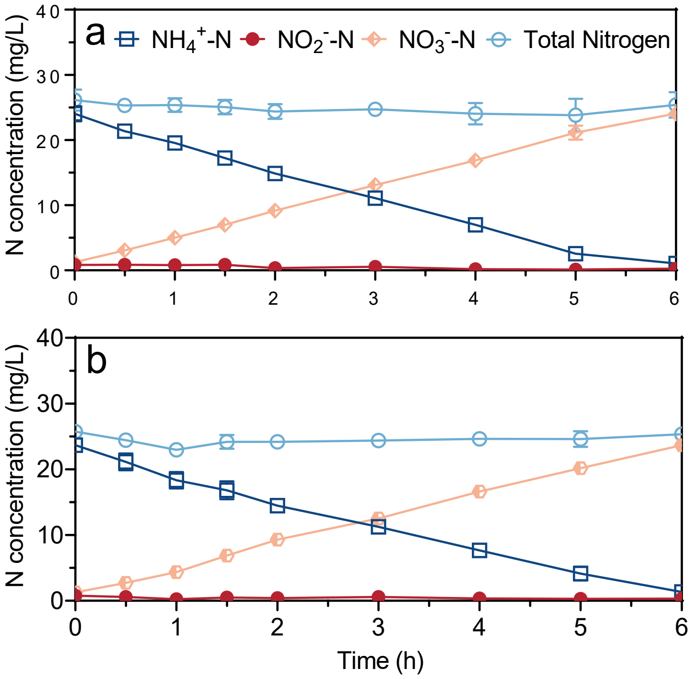

Supplementary Fig. 12. The variations of nitrogenous compounds (NH_4^+-N, NO_3^--N, NO_2^--N and Total Nitrogen) in the aerated activated sludge reactors in the tests with in-sewer E-$FeCO_3$ dosing. Total Nitrogen = NH_4^+-N + NO_3^--N + NO_2^--N. a, Control. **b,** Experiment. Averages of triplicate experiments are reported, with error bars representing standard deviations.

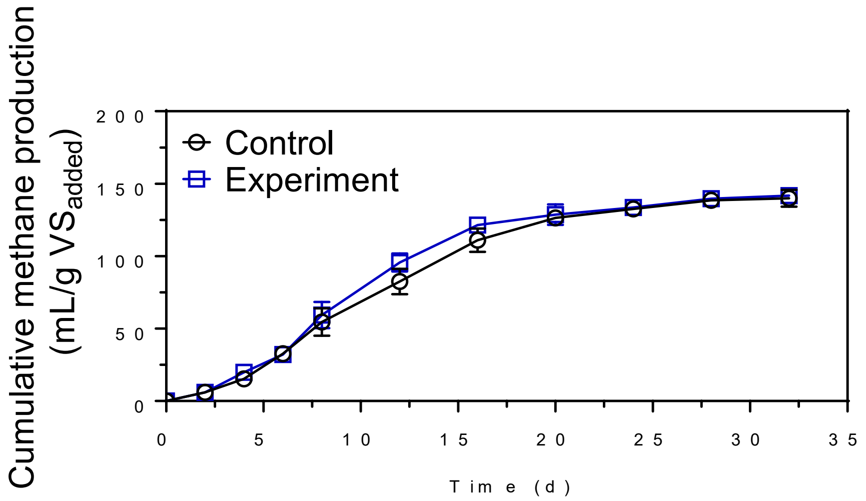

Supplementary Fig. 13. Methane production in the BMP tests with in-sewer dosing of E-$FeCO_3$. Averages of triplicate experiments are reported, with error bars representing standard deviations.

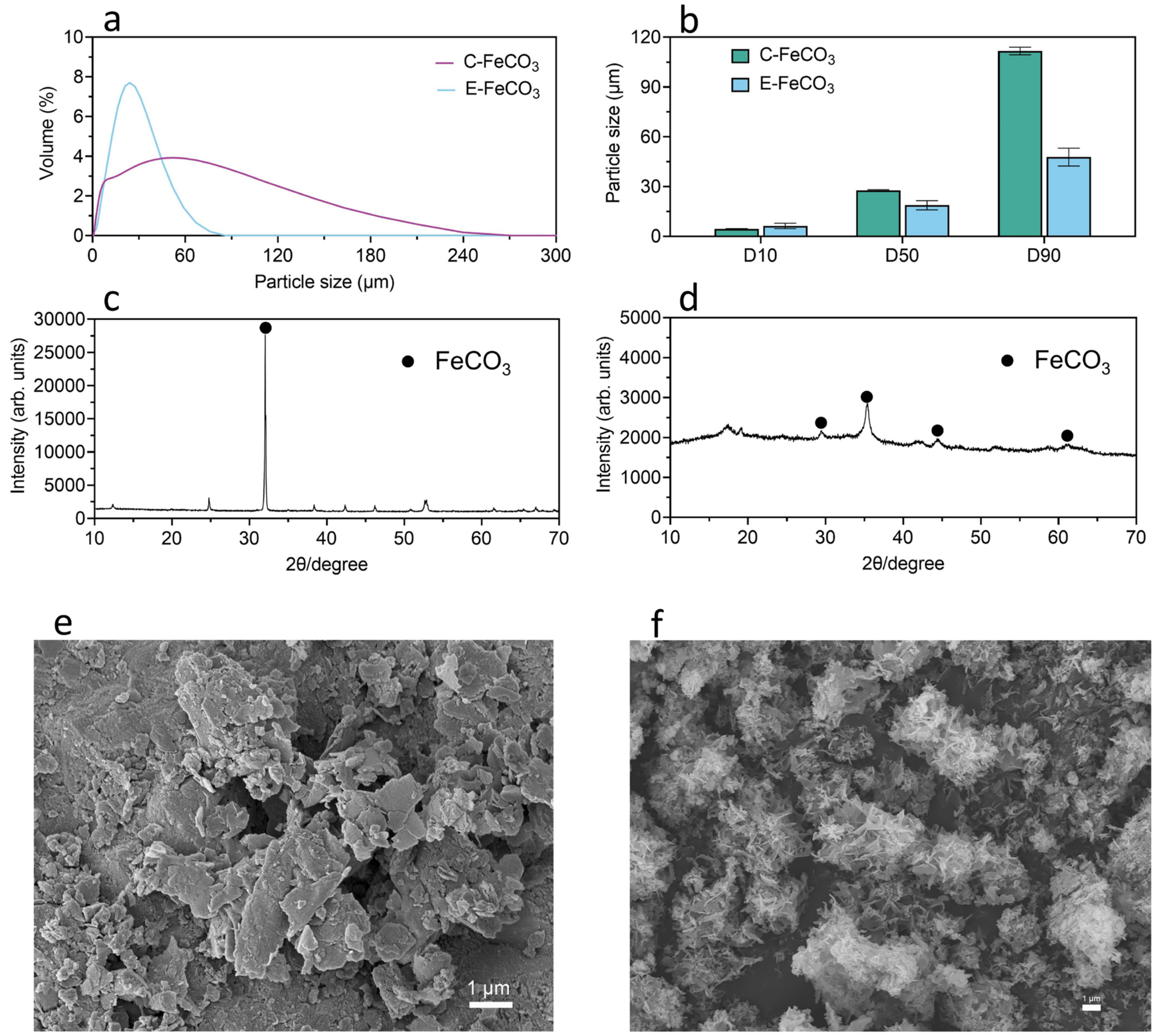

Supplementary Fig. 14. The characteristics of fresh C-$FeCO_3$ and E-$FeCO_3$ (produced in experiments at pH 8.5). a, The particle size distribution. **b,** Values of D_{10}, D_{50}, and D_{90}. **c,** XRD profile of the $FeCO_3$ in the C-$FeCO_3$ slurry. **D**, XRD profile of the $FeCO_3$ in the E-$FeCO_3$ slurry. **e**, SEM micrograph of the C-$FeCO_3$. **f**, SEM micrograph of the E-$FeCO_3$. All values are means ± standard deviations of triplicate tests.

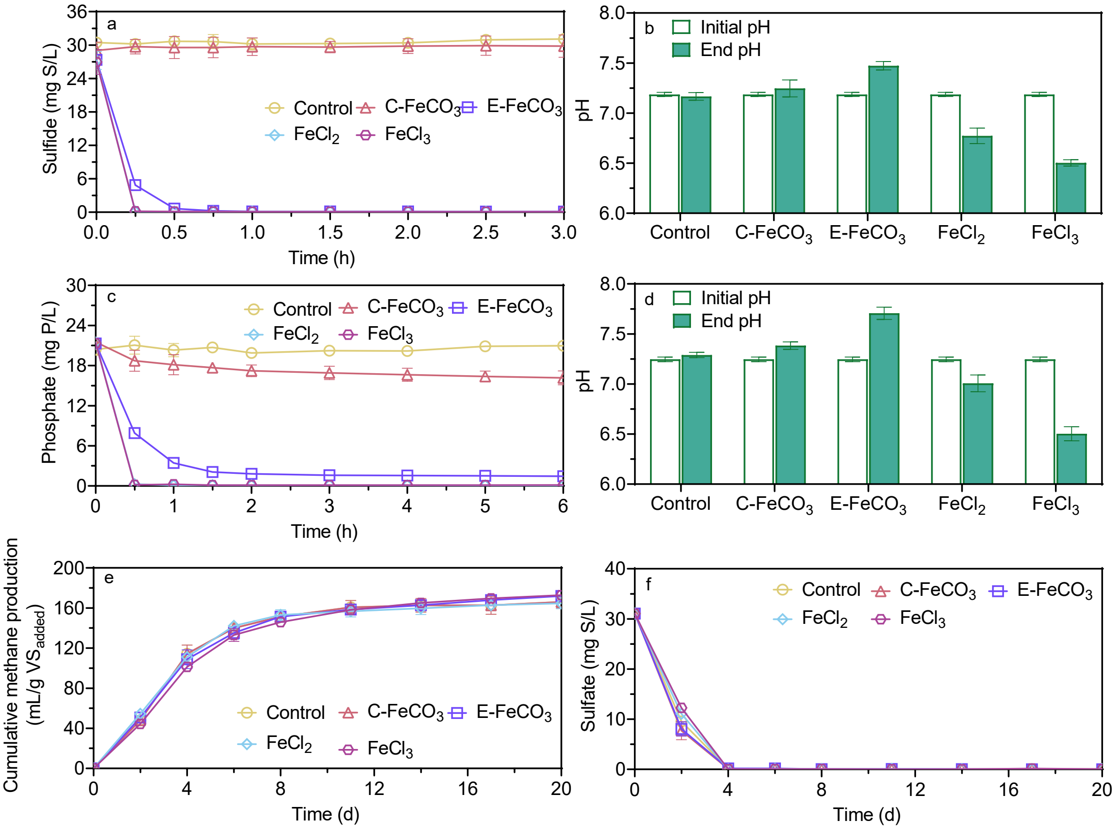

Supplementary Fig. 15. Application of different iron salts to wastewater and sludge management. a, Sulfide control in sewer (overdosing). **b**, pH at the beginning and end of each test in sewer (overdosing). **c**, Phosphate removal in aerated activated sludge (overdosing). **d**, pH at the beginning and end of each test in aerated activated sludge (overdosing). **e**, Methane production in BMP tests. **f**, Sulfate reduction in BMP tests. Averages of triplicate experiments are reported, with error bars representing standard deviations.

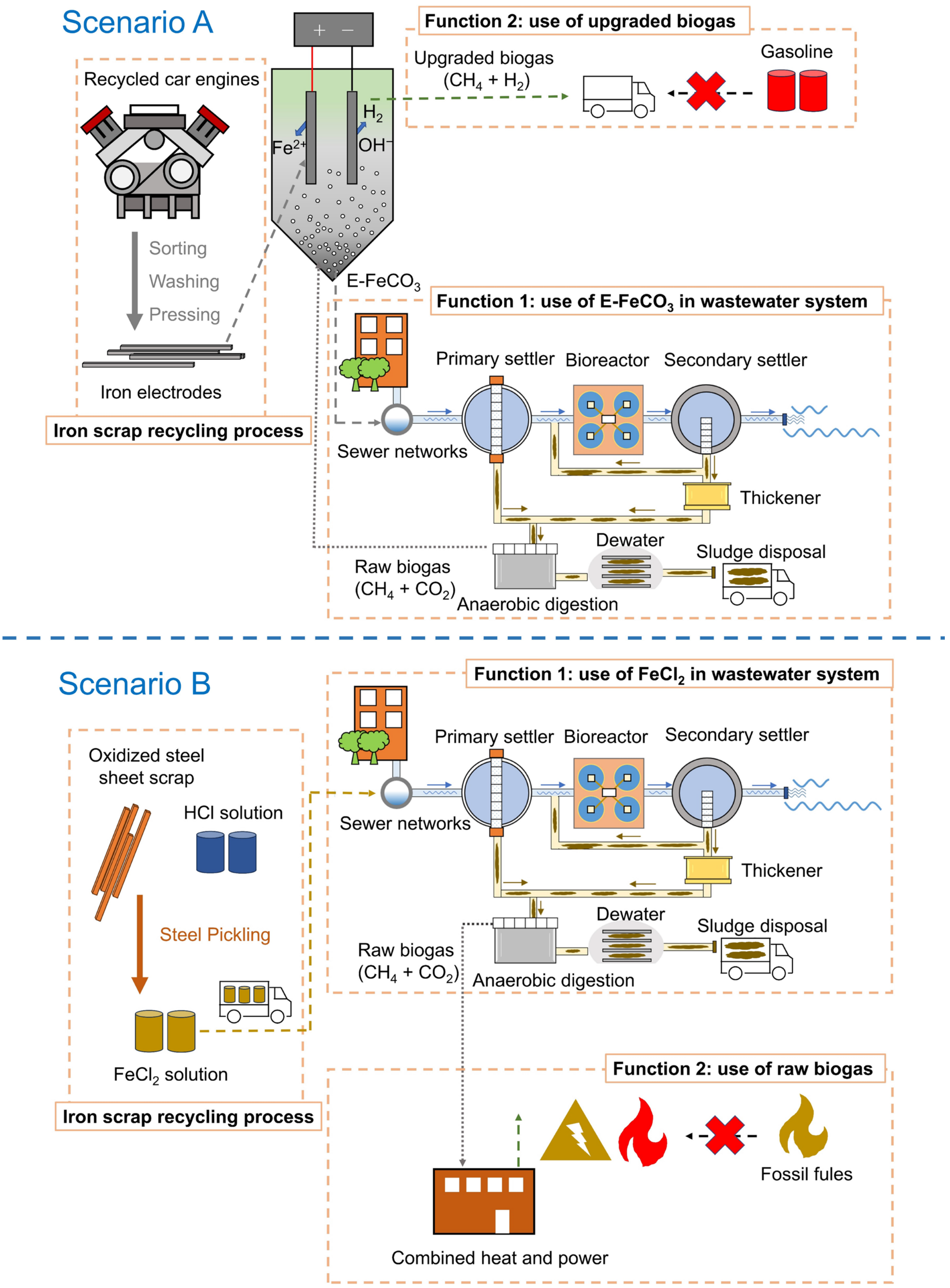

Supplementary Fig. 16. System boundaries and main functions of Scenario A (upper) and Scenario B (below). Scenario A is further divided into A1 and A2 with the electricity for E-FeCO3 production generated from biogas (A1) and from the current mix of primary energy

sources in Australia (A2), respectively. Scenario B is also divided into B1 and B2 with FeCl2 transported for 1,000 km and 4,000 km, respectively.

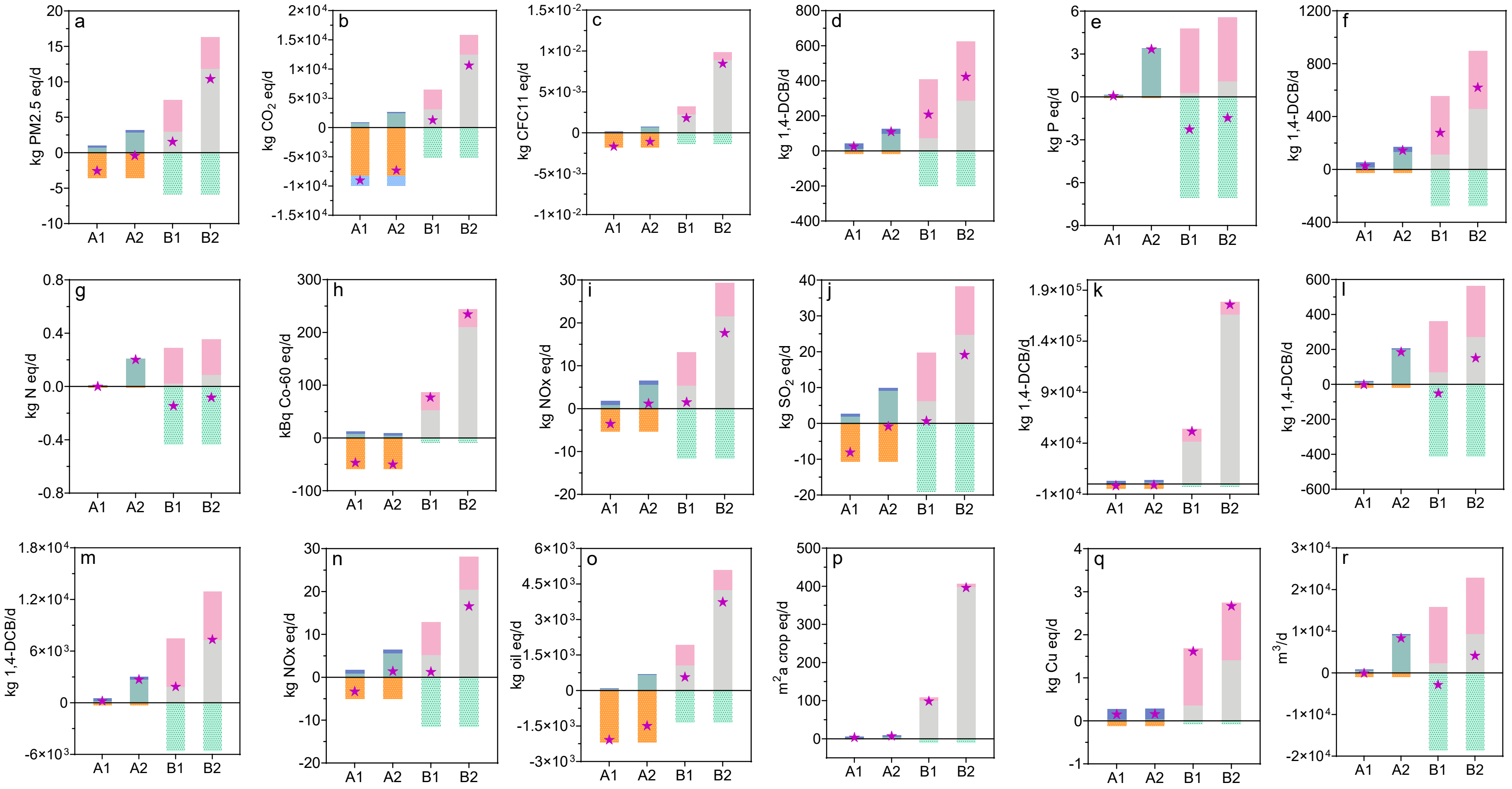

Supplementary Fig. 17. Results of life cycle assessment. a, Fine particulate matter formation. **b,** Global warming. **c,** Stratospheric ozone depletion. **d,** Freshwater ecotoxicity. **e,** Freshwater eutrophication. **f,** Marine ecotoxicity. **g,** Marine eutrophication. **h,** Ionizing radiation. **i,** Ozone formation (terrestrial ecosystems). **j,** Terrestrial acidification. **k,** Terrestrial ecosystems. **l,** Human carcinogenic toxicity. **m,** Human non-carcinogenic toxicity. **n,** Ozone formation (human health). **o,** Fossil resource scarcity. **p,** Land use. **q,** Mineral resource scarcity. **r,** Water consumption.

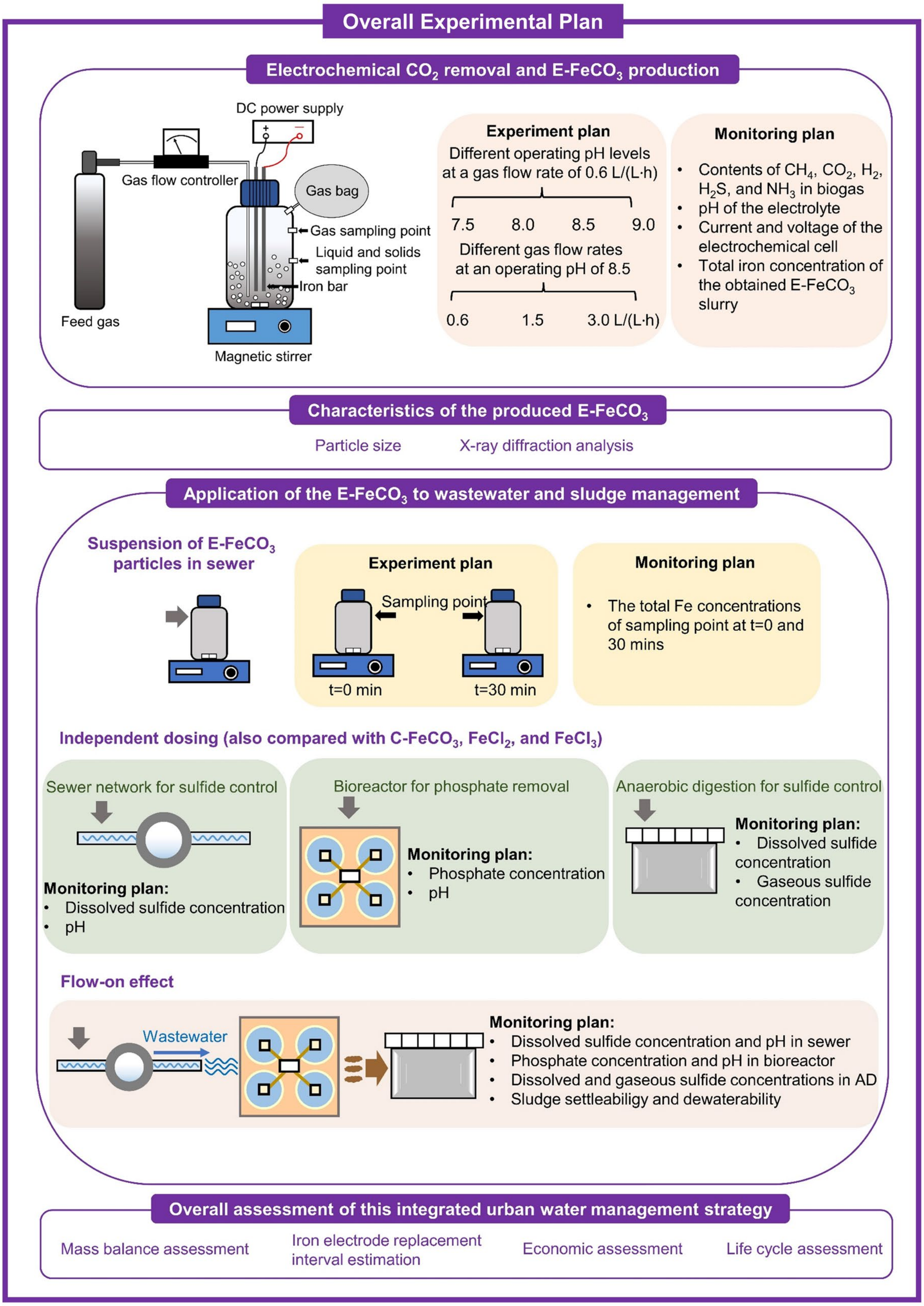

Supplementary Fig. 18. The overall experimental design.

Supplementary Table 1. Summary of results in tests at different pH levels. (All values are means ± standard deviations of triplicate tests)

		Different pH conditions			
		7.5	8.0	8.5	9.0
Feed gas composition (%)	N_2	59.2 ± 0.1			
	CO_2	40.6 ± 0.1			
Outflow gas composition (%)	N_2	59.1 ± 0.4	60.0 ± 0.5	59.5 ± 0.2	60.4 ± 0.6
	H_2	32.5 ± 0.4	34.7 ± 0.2	34.6 ± 0.2	36.1± 0.4
	CO_2	9.1 ± 0.1	6.6 ± 0.5	5.9 ± 0.2	5.0 ± 0.0
CO_2 removal efficiency (%)		76.6 ± 0.2	83.3 ± 1.2	85.1 ± 0.4	87.4 ± 0.1
Total iron concentration (g/L)		6.0 ± 0.1	7.9 ± 0.2	9.2 ± 0.1	12.2 ± 0.1
$R_{Fe/e}$		0.54 ± 0.01	0.55 ± 0.02	0.52 ± 0.01	0.54 ± 0.02
$R_{CO_2/Fe}$		0.87 ± 0.02	0.81 ± 0.02	0.80 ± 0.02	0.53 ± 0.03

$R_{Fe/e}$: The ratio between the total electrons transferred (moles) and the total amount of Fe produced (moles).

$R_{CO_2/Fe}$: The ratio between the total amount CO_2 removed (moles) and the total amount of Fe produced (moles).

Supplementary Table 2. The use of different iron salts in urban wastewater management systems.

	Iron source	Iron dosage	Dosing point	Efficiency	Reference
Dissolved sulfide control in sewer					
	$FeSO_4$	1.47 g Fe/g S	Sewer network	Sulfide removal capacity: 0.59 g S/g Fe	1
	$FeClSO_4$	1.19 g Fe/g S		Sulfide removal capacity: 0.74 g S/g Fe	
	Ferrous salt	2.28 g Fe/g S	Sewer network	Reducing the dissolved sulfide concentration to <0.1 mg S/L	2
	Ferric salt	1.58 g Fe/g S			
	$FeCl_3$	10 mg Fe/L-wastewater	Sewer network	Sulfide removal capacity: 0.43 g S/g Fe	3
	$FeCl_3$	21 mg Fe/L-wastewater	Sewer network	Reducing the dissolved sulfide concentration from 17.1 to <0.2 mg S/L	4
	$FeCl_3$	49 mg Fe/L-wastewater	Sewer network	Sulfide removal capacity: 0.42 g S/g Fe	5
Phosphate control in wastewater treatment system					
	$FeCl_3$	0.57 molar Fe/molar P	Wastewater treatment system	P removal capacity: ~0.49 g P/g Fe	6
		1.08 molar Fe/molar P		P removal capacity: ~0.36 g P/g Fe	

		1.48 molar Fe/molar P		P removal capacity: ~0.30 g P/g Fe	
		2.85 molar Fe/molar P		P removal capacity: ~0.09 g P/g Fe	
	$Fe(OH)_3$	3.86 molar Fe/molar P		P removal capacity: ~0.10 g P/g Fe	
		> 4.80 molar Fe/molar P		P removal capacity: <0.09 g P/g Fe	
	$FeCl_3$	35 mg Fe/L-wastewater		Reducing the P concentration from ~6.7 to ~0.1 mg P/L	7
	$FeCl_3$	20 mg Fe/L-wastewater		Reducing the P concentration from to ~7.1 to ~0.3 mg P/L	8
	$FeCl_2$	20 mg Fe/L-wastewater	Sewer network (Evaluating the flow-on effect on wastewater treatment system)	P removal capacity: 0.37 g P/g Fe	9
	$FeCl_3$	20 mg Fe/L-wastewater		P removal capacity: 0.44 g P/g Fe	
	$FeCl_3$	10 mg Fe/L-wastewater	Sewer network (Evaluating the flow-on effect on wastewater treatment system)	P removal capacity: 0.47 g P/g Fe	3
Dissolved and gaseous sulfide control in anaerobic digestion					
	$FeCl_3$	7.40—9.43 mg Fe/g TS	Anaerobic digestion	H_2S removal efficiency of ~93%	10

	$FeCl_3$	4.5 mg Fe/g TS		H_2S removal efficiency of ~87%	11
	$FeCl_3$	5—20 mg Fe/L-wastewater	Sewer network (Evaluating the flow-on effect on anaerobic digestion)	Reducing the dissolved sulfide concentration to <0.2 mg S/L	12
	$FeCl_3$	10 mg Fe/L-wastewater	Sewer network (Evaluating the flow-on effect on anaerobic digestion)	• Reducing the dissolved sulfide concentration from ~23.7 to ~2.7 mg S/L (~88.5% removal efficiency)<br>• Reducing the H_2S concentration in biogas from ~911 to ~130 ppmv (~82.3% removal efficiency)	3
Sludge settleability					
	$FeCl_3$	10 mg Fe/L-wastewater	Sewer network (Evaluating the flow-on effect on wastewater treatment system)	Reducing the SVI from ~75 to ~55 mL/g	3
	$FeCl_3$	20 mg Fe/L-wastewater	Wastewater treatment system	Reducing the SVI from ~78 to ~45 mL/g	8
	$FeCl_3$	35 mg Fe/L-wastewater	Wastewater treatment system	Reducing the SVI from ~105 to ~45 mL/g	7
Sludge dewaterability					

	$FeCl_3$	35 mg Fe/L-wastewater	Wastewater treatment system	Reducing the SRF from ~2.1×10^{13} to ~5.0×10^{12} m/kg	7
	$FeCl_3$	10 mg Fe/L-wastewater	Sewer network<br>(Evaluating the flow-on effect on anaerobic digestion)	Increasing the sludge dewaterability from ~15.9 to ~19.4%	3
	$FeCl_3$	20 mg Fe/L-wastewater	Wastewater treatment system	Reducing the SRF from ~2.3×10^{13} to ~3.4×10^{12} m/kg	8

Supplementary Table 3. Sulfide removal capacity with E-$FeCO_3$ produced in three different influent scenarios.

	General parameters	Unit	Low carbon	Medium carbon	High carbon
Mass balance analysis					
	Wastewater loading rate	m^3/d		120,000	
	Influent biodegradable COD concentration	g/m^3	300	500	800
	Biogas volume to mole conversion factor	$mole/m^3$		40[1]	
	Daily production of methane (assuming 7% of influent bCOD is converted to methane)	m^3/day	984	1,641	2,625
	Daily biogas production (containing methane at 60%)	m^3/day	1,641	2,734	4,375
	Daily CO_2 production (40% of biogas)	m^3/day	656	1,094	1,750
	Daily E-$FeCO_3$ production capacity[2]	kg Fe/day	1,470	2,450	3,920
	Achievable E-$FeCO_3$ concentration if all dosed to the catchment	g Fe/m^3	12.3	20.4	32.7
	Sulfide removal capacity[3]	g S/ m^3	**6.1**	**10.2**	**16.3**
Economic assessment					
Main input material costs					
	Electricity[4]	A$/y	154,074	256,791	410,865
	Current needed[5]	A	58,628	97,713	156,341
	Voltage to be applied[6]	V		1.5	

		Power consumption	kWh/y	770,372	1,283,954	2,055,326
	NaCl[7]			358	596	954
	Iron[8]		A$/y	378,741	631,235	1,009,976
	Biogas[9]			107,789	179,648	287,438
	Total			**640,962**	**1,068,270**	**1,709,233**
Value of products						
	E-$FeCO_3$[10]			1,509,047	2,515,078	4,024,125
		Price of $FeCl_2$ solution[11]	A$/ton		350	
		Price of Fe in $FeCl_2$ solution[12]	A$/ton Fe		2800	
	Upgraded biogas as a transport fuel[13]		A$/y	573,478	955,796	1,529,274
		Energy in upgraded biogas[14]	MJ/d	42,565	71,094	113,750
		Gasoline replaced[15]	L/d	1,209	2,014	3,223
	Total		A$/y	**2,082,525**	**3,470,874**	**5,553,399**

[1]Assuming 25 ℃. [2]Assuming complete CO_2 removal, and one mole Fe is required for each mole of CO_2 removed. [3]Assuming a sulfide removal capacity of 0.5 g S/g Fe. [4]Assuming an electricity price of A$0.2/kWh. [5]Two moles of electrons per mole of Fe oxidized. [6]The reaction takes place at 0.39V theoretically. Assuming 1.5V by considering some minor loss at a low current density. [7]Estimation based on a NaCl price of 50 A$/t and an obtained E-$FeCO_3$ slurry concentration of 150 g Fe/L. [8]Assuming recycled iron is used at A$600/ton. [9]Biogas is used to replace natural gas for power production; the price of natural gas is A$0.3/$m^3$. [10]As E-$FeCO_3$ can be a substitute of $FeCl_2$, so the value of E-$FeCO_3$ is defined based on the cost of $FeCl_2$ it replaces. [11]Quotation from a supplier. [12]The $FeCl_2$ solution contents Fe at 12% w/w. [13]This is defined as the price of replaced gasoline (assuming A$1.3/L). [14]This is the sum of energy in CH_4 (assuming 36 MJ/m^3) and energy in H_2 (assuming 11 MJ/H_2; H_2 production is equal to CO_2 removal). [15]Assuming 8.5L/100km, which is equivalent to 300 MJ/100 km, as assumed for gas; 45 MJ/kg gasoline or 36 MJ/L gasoline (gasoline density 0.8 kg/L).

Supplementary Table 4. Estimation of the iron plate replacement interval.

	Unit	Low carbon	Medium carbon	High carbon
Influent biodegradable COD concentration	g/m^3	300	500	800
Daily CO_2 production[1]	m^3/day	656	1094	1750
Daily Fe consumption[1]	kg Fe/day	1,470	2,450	3,920
Volume of electrochemical cell	m^3		200	
Required CO_2 mass transfer efficiency (K_La)[2]	/d	206	343	548
Iron plate spacing	cm		1	
Iron plate thickness	cm		1	
Volume of iron electrode when new[3]	m^3		67	
Mass of electrode when new[4]	kg		522,600	
Iron electrode replacement interval[5]	day	302	181	113

[1]See calculation in Supplementary Table 2. [2]Assuming CO_2 accounts for 5% of the upgraded biogas. [3]Assuming the volume of iron electrode is 1/3 of the total volume. [4]Assuming iron density of 7800 kg/m^3. [5]Assuming replacement occurs at 85% consumption.

Supplementary Table 5. The detection methods used in this study.

Parameter	Detection method
MLSS and MLVSS	1. Dry a glass fiber filter (0.22 μm) and then weigh it.<br>2. Extract 5 mL mixed liquid and then filter it using the pre-dried and pre-weighed glass fiber filter.<br>3. Dry the filter and its contents in an oven at a temperature of 105 °C for 24 h.<br>4. Weigh the filter and dried solids.<br>5. Calculate MLSS using the formula: MLSS (mg/L) = (Weight of solids on filter (mg) – the weight of pre-dried filter (mg)) / Volume of mixed liquor sample (L).<br>6. After obtaining the MLSS weight, transfer the filter and sludge to a crucible with a lid, and then weigh it.<br>7. Place the crucible with the lid in a muffle furnace and heat it at a temperature 550 °C for two hours to combust all the organic matter.<br>8. Cool down the crucible in a desiccator and then weigh it. Calculate MLVSS using the formula: MLVSS (mg/L) = (Weight of crucible before combustion (mg) – Weight of crucible after combustion (mg)) / Volume of mixed liquor sample (L).
TS and VS	1. Dry a crucible and then weigh it.<br>2. Add a certain amount of sludge to pre-dried crucible, and then weigh it again (The increased weight compared to the clean crucible represents the added volume of sludge).<br>3. Dry the crucible and the sludge in an oven at a temperature of 105°C for 24 h.<br>4. Weigh the filter and dried solids.<br>5. Calculate TS using the formula: TS (g/L) = (Weight of solids on filter (g) – the weight of pre-dried filter (g)) / Volume of mixed liquor sample (L).<br>6. After obtaining the TS weight, transfer the filter and sludge to a crucible with a lid, and then weigh it.<br>7. Place the crucible with the lid in a muffle furnace and heat it at a temperature 550 °C for two hours to combust all the organic matter.<br>8. Cool down the crucible in a desiccator and then weigh it. Calculate VS using the formula: VS (g/L) = (Weight of crucible before combustion (g) – Weight of crucible after combustion (g)) / Volume of mixed liquor sample (L).
SVI	1. Collect 105 mL mixed liquid from the bioreactor, 5 mL of mixed liquid was used for the determination of MLSS concentration. The residual 100 mL was added to a clean and dry measuring cylinder.<br>2. After settling for 30 min, record the volume of the settled sludge.<br>3. Calculate the SVI using the following formula: SVI (mL/g) = volume of settled sludge (mL) / (volume of mixed liquor sample (mL) × sludge concentration (MLSS) (g/L))
TCOD and SCOD	1. Liquid sample (filtered sample for SCOD) of 3 mL were added to the glass tube provided by the supplier (Merck, Germany).<br>2. The liquid sample was digested at 148 °C for 2 h.<br>3. After that, the COD concentration was determined by a spectrophotometer.
Gaseous CH4, CO2 and H2	1. Gas sample of 100μL was collected with a gastight syringe (1710SL, Hamilton, USA), and then injected into a gas chromatograph for CH_4, CO_2, and H_2 concentrations determination.<br>2. The Agilent gas chromatograph (GC, 7890A, Agilent, USA) was equipped with a HayeSepQ column (2440 × 2.0mm) and a thermal conductivity detector. Argon, at a flow rate of 28 mL/min, was supplied as the carrier gas. During the test process, the injector, column, and detector temperatures were controlled at 110, 45, and 170 °C, respectively. Different types of gas were identified according to their unique retention times, and their concentrations were calculated according to the standard curve.
Total Fe	1. The slurry of 2 mL was extracted from the reactor and immediately added to a solution of 1M HCl of 10 mL.<br>2. After that, 0.2 mL of solution obtained in Step 1, 0.8 mL of 1M HCl and 1 mL of 1,10-phenanthroline (1 g/L) were mixed in a 2 mL tube.<br>3. The absorbency of the solution obtained in Step 2 was determined by a spectrophotometer, and their concentrations were calculated according to the

	standard curve.
SRF	SRF=$\frac{2PA^2b}{\mu W}$ A filtration test was performed to determine the SRF. In this test, the filtration pressure (P) (N/m^2) was 0.1 N/m^2, filter area (A) (m^2) was 38.485 cm^2, and viscosity of the filtrate (μ) (N s/m^2) was assumed to be 1.0087 N s/m^2. About 50 mL of the sludge was added to the suction filter, and the supernatant was gradually filtered out under the pressure. The volume of supernatant was recorded every 2 seconds until the supernatant is difficult to be filtered out. After filtration, the concentration of the residual sludge was measured, which is w (kg/m^3). In addition, the slope of filtrate discharge curve (b) was determined through linear regression of the linear part of the volume of supernatant against filtration time obtained from the filtration test.

MLSS: mixed liquor suspended solids; MLVSS: mixed liquor volatile suspended solid; TS: total solid; VS: volatile solid; SVI: sludge volume index; TCOD: total chemical oxygen demand; SCOD: soluble chemical oxygen demand; SRF: specific resistance to filtration.

Supplementary Table 6. Processes modelled in LCA.

Process	Description
Iron scrap recycling	The iron scrap, one common recycled material, is collected, sorted, and processed for reuse in the electrochemical process. The transportation is usually within the city, with a short transport. Thus, the environmental impact of this transportation is neglected.
Electrochemical process	The process produces two products, namely E-$FeCO_3$ for wastewater management replacing $FeCl_2$, and upgraded biogas as car fuel replacing gasoline. Iron scraps and the biogas are two main raw materials. Electricity and water are consumed by the process, which are also considered in the assessment.
Upgraded biogas utilization	H_2 is produced stoichiometrically while CO_2 is removed from biogas by the electrochemical process. The upgraded biogas is utilized as a transport fuel. Hydrogen-enriched compressed natural gas (HCNG) powered vehicles are predicted to be widely applied in the future. The average fuel consumption of HCNG-powered vehicles is rarely reported, the methane and hydrogen consumption data for natural gas- and hydrogen-powered vehicles are used instead.<br>The use of upgraded biogas as a transport fuel substitutes gasoline. The equivalent substitution is calculated based on an equivalent travel distance. The life cycle of the gasoline is modeled from energy production, distribution, to energy conversion in the vehicle (i.e. driving). Based on the Australian gasoline statistics, around 75% of gasoline consumed is imported, which is from Malaysia (42%), United Arab Emirates (21%), Indonesia (13%), Gabon (9%), Brunei Darussalam (8%), and New Zealand (7%). The transport distance of gasoline is simply counted based on the straight-line distances between Australia and the import states. For inventory from vehicles driving, CO_2 emission is counted, and other exhaust emissions are not included in this study.
$FeCl_2$ production and transportation	$FeCl_2$ is produced as a waste product from steel pickling in Australia. The typical transport distance ranges from several hundred to two thousand kilometres, so here we use 1000 km as an average for the current practice. A distance of 4000 km represents the longest transportation distance in Australia.
Biogas utilization	It is assumed biogas, in Scenario B, is used for combined heat and power production, with energy efficiencies of 45% and 35%, respectively. It is assumed that 100% power and 35% heat are effectively used. Biogas thus substitutes the electricity mainly produced from fossil energy.

Supplementary Table 7. LCA model parameters.

Category	Parameters*	Units	Values
General parameters	Wastewater loading rate	m^3/d	120,000
	Influent biodegradable COD concentration	g/m^3	500
	Fraction of wastewater biodegradable COD converted to methane	%	7
	Volume to mole conversion factor	$mole/m^3$	40
	Methane fraction in biogas	%	60
	Carbon dioxide fraction in biogas	%	40
Scenario A parameters	Electricity consumption in electrochemical process	kWh/kg Fe	1.5
	CO_2 removal efficiency	%	90
	Average fuel consumption of natural gas powered vehicles	kg/100 km	5.27
	Average fuel consumption of hydrogen powered vehicles	kg/100 km	1.0
	Average fuel consumption of gasoline powered vehicles	kg/100 km	6.63
	Transportation distance of purchased gasoline	km	4774
	CO_2 emission factor of gasoline combustion	kg/kg	2.06
Scenario B parameters	Active ingredient (i.e. Fe) of $FeCl_2$ product	%	12
	The transport distance of purchased $FeCl_2$	km	**1000 (B-1)**<br>**4000 (B-2)**
	Efficiency of heat production	%	45
	Efficiency of power production	%	35
	Conversion coefficient between heat and power	kJ/kWh	7350
	Utilization factor of heat	%	35
	Utilization factor of power	%	100

* For uncertainty analysis, most parameters included in Table 5 are simply given a variation of ±10%, except for two parameters in Scenario B. The transport distance of purchased $FeCl_2$ is given a broad variation from 500 km to 4000 km. Utilization factors of heat and power in baseline is set as the maximum values in status quo with the minimum values of 0%, and 80%, respectively.

Supplementary Table 8. The average results of 10,000 Monte Carlo simulations.

Indictors	Units	Scenario A1	Scenario A2	Scenario B1	Scenario B2
Fine particulate matter formation	kg $PM_{2.5}$ eq	-2.58E+00	-4.14E-01	2.66E+00	1.16E+01
Fossil resource scarcity	kg oil eq	-2.10E+03	-1.50E+03	8.22E+02	4.04E+03
Freshwater ecotoxicity	kg 1,4-DCB	2.53E+01	1.10E+02	2.47E+02	4.66E+02
Freshwater eutrophication	kg P eq	6.41E-02	3.36E+00	-9.98E-01	-1.88E-01
Global warming	kg CO_2 eq	-9.08E+03	-7.37E+03	2.24E+03	1.17E+04
Human carcinogenic toxicity	kg 1,4-DCB	-1.73E+00	1.87E+02	2.41E+01	2.29E+02
Human non-carcinogenic toxicity	kg 1,4-DCB	1.94E+02	2.71E+03	2.92E+03	8.46E+03
Ionizing radiation	kBq Co-60 eq	-5.24E+01	-5.05E+01	7.97E+01	2.39E+02
Land use	m^2a crop eq	3.21E+00	6.92E+00	1.02E+02	4.04E+02
Marine ecotoxicity	kg 1,4-DCB	2.68E+01	1.45E+02	3.32E+02	6.79E+02
Marine eutrophication	kg N eq	-2.16E-04	2.03E-01	-6.76E-02	-3.34E-03
Mineral resource scarcity	kg Cu eq	1.51E-01	1.59E-01	1.64E+00	2.71E+00
Ozone formation (Human health)	kg NO_x eq	-3.35E+00	1.41E+00	3.40E+00	1.89E+01
Ozone formation (Terrestrial ecosystems)	kg NO_x eq	-3.56E+00	1.21E+00	3.67E+00	2.00E+01
Stratospheric ozone depletion	kg CFC_{11} eq	-1.69E-03	-1.09E-03	2.09E-03	8.83E-03

Terrestrial acidification	kg SO_2 eq	-8.14E+00	-8.35E-01	4.19E+00	2.29E+01
Terrestrial ecotoxicity	kg 1,4-DCB	-1.77E+03	-1.04E+03	5.26E+04	1.79E+05
Water consumption	m^3	-7.97E+01	8.41E+03	5.56E+02	7.63E+03

Reference

1 Tomar, M. & Abdullah, T. H. A. Evaluation of chemicals to control the generation of malodorous hydrogen sulfide in waste water. *Water Res.* **28**, 2545-2552 (1994). https://doi.org:https://doi.org/10.1016/0043-1354(94)90072-8

2 Firer, D., Friedler, E. & Lahav, O. Control of sulfide in sewer systems by dosage of iron salts: Comparison between theoretical and experimental results, and practical implications. *Sci. Total Environ.* **392**, 145-156 (2008). https://doi.org:https://doi.org/10.1016/j.scitotenv.2007.11.008

3 Rebosura Jr, M. *et al.* A comprehensive laboratory assessment of the effects of sewer-dosed iron salts on wastewater treatment processes. *Water Res.* **146**, 109-117 (2018).

4 Zhang, L., Keller, J. & Yuan, Z. Inhibition of sulfate-reducing and methanogenic activities of anaerobic sewer biofilms by ferric iron dosing. *Water Res.* **43**, 4123-4132 (2009).

5 Cao, J., Zhang, L., Hong, J., Sun, J. & Jiang, F. Different ferric dosing strategies could result in different control mechanisms of sulfide and methane production in sediments of gravity sewers. *Water Res.* **164**, 114914 (2019).

6 Thistleton, J., Berry, T.-A., Pearce, P. & Parsons, S. Mechanisms of chemical phosphorus removal II: iron (III) salts. *Process Saf. Environ. Prot.* **80**, 265-269 (2002).

7 Hu, Z. *et al.* Centralized iron-dosing into returned sludge brings multifaceted benefits to wastewater management. *Water Res.* **203**, 117536 (2021). https://doi.org:https://doi.org/10.1016/j.watres.2021.117536

8 Hu, Z. *et al.* Novel Use of a Ferric Salt to Enhance Mainstream Nitrogen Removal from Anaerobically Pretreated Wastewater. *Environ. Sci. Technol.* **57**, 6712-6722 (2023).

9 Gutierrez, O., Park, D., Sharma, K. R. & Yuan, Z. Iron salts dosage for sulfide control in sewers induces chemical phosphorus removal during wastewater treatment. *Water Res.* **44**, 3467-3475 (2010). https://doi.org:https://doi.org/10.1016/j.watres.2010.03.023

10 Akgul, D., Abbott, T. & Eskicioglu, C. Assessing iron and aluminum-based coagulants for odour and pathogen reductions in sludge digesters and enhanced digestate dewaterability. **598**, 881-888 (2017).

11 Abbott, T. & Eskicioglu, C. Effects of metal salt addition on odor and process stability during the anaerobic digestion of municipal waste sludge. *Waste Manage.* **46**, 449-458 (2015). https://doi.org:https://doi.org/10.1016/j.wasman.2015.07.050

12 Ge, H., Zhang, L., Batstone, D. J., Keller, J. & Yuan, Z. Impact of iron salt dosage to sewers on downstream anaerobic sludge digesters: sulfide control and methane production. *J. Environ. Eng.* **139**, 594-601 (2013).
